# Quantifying the links between land use and population growth rate in a declining farmland bird

**DOI:** 10.1002/ece3.4766

**Published:** 2019-02-05

**Authors:** Matthieu Paquet, Debora Arlt, Jonas Knape, Matthew Low, Pär Forslund, Tomas Pärt

**Affiliations:** ^1^ Department of Ecology Swedish University of Agricultural Sciences Uppsala Sweden

**Keywords:** conservation, farmland birds, habitat management, habitat quality, integrated population model, land use, *Oenanthe oenanthe*, population dynamics

## Abstract

Land use is likely to be a key driver of population dynamics of species inhabiting anthropogenic landscapes, such as farmlands. Understanding the relationships between land use and variation in population growth rates is therefore critical for the management of many farmland species. Using 24 years of data of a declining farmland bird in an integrated population model, we examined how spatiotemporal variation in land use (defined as habitats with “Short” and “Tall” ground vegetation during the breeding season) and habitat‐specific demographic parameters relates to variation in population growth taking into account individual movements between habitats. We also evaluated contributions to population growth using transient life table response experiments which gives information on contribution of past variation of parameters and real‐time elasticities which suggests future scenarios to change growth rates. LTRE analyses revealed a clear contribution of Short habitats to the annual variation in population growth rate that was mostly due to fledgling recruitment, whereas there was no evidence for a contribution of Tall habitats. Only 18% of the variation in population growth was explained by the modeled local demography, the remaining variation being explained by apparent immigration (i.e., the residual variation). We discuss potential biological and methodological reasons for high contributions of apparent immigration in open populations. In line with LTRE analysis, real‐time elasticity analysis revealed that demographic parameters linked to Short habitats had a stronger potential to influence population growth rate than those of Tall habitats. Most particularly, an increase of the proportion of Short sites occupied by Old breeders could have a distinct positive impact on population growth. High‐quality Short habitats such as grazed pastures have been declining in southern Sweden. Converting low‐quality to high‐quality habitats could therefore change the present negative population trend of this, and other species with similar habitat requirements.

## 1 INTRODUCTION

Human land use and management have profound effects on populations and biodiversity. In particular, agricultural ecosystems are suffering from a dramatic biodiversity crisis, with many farmland bird species in Europe and North America displaying sharp declines during the last 50 years (Donald, Green, & Heath, 2001; Inger et al., 2014; Stanton, Morrissey, & Clark, 2018; Vickery et al., 2013). Agricultural intensification is a suggested major cause of biodiversity declines because of specific drivers such as increased use of pesticides and fertilizers, changed land use and crop types, loss of remnant habitats (e.g., hedges and shrubs, rough grass strips), and intensified farming practices (e.g., Newton, 2004; Emmerson et al., 2016). However, knowledge on the direct links between these agricultural drivers and population demographic rates is largely lacking as most studies are based on large‐scale correlations between potential drivers and population numbers or investigate only a limited set of demographic rates (Morrison, Robinson, Butler, Clark, & Gill, 2016). Without knowing the demographic drivers of population trends, any strategies to mitigate species at risk will be highly uncertain and sometimes not result in the expected positive change in population numbers (Kleijn & Sutherland, 2003).

Different land use types will likely vary in breeding habitat quality because demographic traits and individual movement patterns for farmland birds often differ between farmland habitats. Populations inhabiting heterogeneous landscapes should therefore display habitat‐dependent dynamics (e.g., Pulliam, 1988; Pulliam & Danielson, 1991; Holmes, Marra, & Sherry, 1996), where an understanding of how different habitat types influence the different demographic rates and population growth will be integral for a deeper understanding of how land management practices impact population growth.

A central question is how much of the observed dynamics of a population can be attributed to breeding habitat (i.e., land use) and its effects on demography? Previous analyses that aim to assess habitat quality and its influence on population changes in farmland birds have mainly examined habitat‐specific variation in a single demographic trait or in local abundance (Newton, 2004) and have generally not accounted for the effects of immigration. However, to predict population responses to land use change, we need to investigate the full suite of demographic rates and their relationship to habitat types. In specific, we need to know (a) whether a habitat‐dependent change in a given demographic trait may be compensated by other demographic traits (Matter & Roland, 2012) and (b) the extent to which habitat occupancy patterns together with immigration rates contribute to variation in local population growth rate.

Here, we use an integrated population model (IPM; Besbeas, Freeman, Morgan, & Catchpole, 2002; Schaub & Abadi, 2011) combining abundance, reproductive data, and multistate capture–recapture data (McCrea et al., 2010; Péron, Crochet, Doherty, & Lebreton, 2010; Weegman et al., 2016) to fully quantify the relative importance of contrasting habitats for variation in demography and total population growth rate. We investigated the contribution of two contrasting farmland habitat types (determined by farmland land use) on annual variation in population growth rate using long‐term demographic data of a declining farmland passerine, the northern wheatear (*Oenanthe oenanthe*). Previously, we have documented strong land use‐specific effects on demographic rates that could be captured by grouping land uses into two contrasting habitat types described by their vegetation structure, that is, “Short” habitats with permanently Short or sparse ground vegetation during the breeding season and “Tall” habitats with ground vegetation growing Tall (Pärt 2001; Arlt, Forslund, Jeppsson, & Pärt, 2008). These results were based on habitat‐specific population growth rates, whereas the effect of habitat and habitat use on total population growth rate was not investigated. Here, we quantify the effect of habitats on the variation in total population growth rate by integrating habitat‐specific occupancy, transitions, demography, and immigration.

Specifically, we integrate several data sources to investigate (a) habitat‐specific demographic rates and whether there exist compensation among these demographic rates and (b) the relative contribution of habitat‐specific demographic drivers, including habitat occupancy and apparent immigration, to past variation in annual population growth rates (using transient life table response experiments; Koons, Arnold, & Schaub, 2017). Because habitat‐specific dynamics and contributions to population growth may change over time, for example, depending on environmental and/or demographic variation, we also investigated whether habitat‐specific contributions of specific parameters to variation in population growth, including habitat occupancies, changed across the study period of 24 years. Last, we examined which parameters have greatest potential to change and possibly increase the population growth rate (using real‐time elasticity analysis; Haridas, Tuljapurkar, & Coulson, 2009).

## 2 MATERIALS AND METHODS

### 2.1 Study system and habitat

The northern wheatear is a small insectivorous migratory passerine bird species that breeds in Europe, Asia, and North America and overwinters in sub‐Saharan Africa. As wheatears are ground‐foraging and mostly ground‐nesting birds, ground vegetation (i.e., field layer) height is a crucial component of habitat quality. Short field layers have been associated with higher prey availability (Tye, 1992), lower nest predation risk (Pärt 2001; Schneider, Low, Arlt, & Pärt, 2012), and higher adult survival (Low, Arlt, Eggers, & Pärt, 2010) compared to sites with Tall field layers. Our study area (60 km^2^) is located in a heterogeneous agricultural landscape, southeast of Uppsala in southern central Sweden (59,500 N, 17,500 E). Short sites have a permanently Short (≤5 cm) field layer throughout the breeding season on at least 0.25 ha (i.e., the minimum territory size) within 50 m of the nest site while Tall sites have a growing field layer reaching a height of more than 5 cm at the time of nestling feeding. Short and Tall habitats are linked to agricultural land use (Short: e.g., farmyards and grazed pastures, Tall: e.g., spring‐ and autumn‐sown crop fields, ungrazed pastures, and unmanaged grasslands), with these habitat types being very distinct and displaying habitat‐specific demography suggesting that Tall habitats are demographic sinks and Short habitats may act as sources (Arlt et al., 2008). In our study area, about 230 territory sites have been occupied by wheatears at least once since 1993 when the yearly monitoring started. About 100–180 pairs breed in the area per year.

For each territory site, we record the identity of the breeding pairs (sex, age‐class, and if present, individual color‐ring combination), habitat type (Short or Tall), and more detailed demographic data in a smaller central part of the study area (~40 km^2^, 179 territory sites, 70–90 pairs per year).

### 2.2 Demographic data

We used individual‐based, age‐class‐ and habitat‐specific data collected from 1993 to 2016 on breeding success, number of fledglings produced, recruitment rate of juveniles (resighting of fledglings as one‐year‐Old breeders), apparent survival of adults (adult resighting as breeders), and the number of breeding territories occupied.

#### 2.2.1 Breeding success

We included data from all occupied sites with records of the fate of nests (i.e., success or failure) in the total study area (60 km^2^). We determined breeding attempts to be successful if we had observed one or more fledged chicks or heard intense warning calls from parents at the time of fledging (Young ≥ 15 days Old). At a site, breeding was considered “successful” if at least one fledgling was produced the given year and “failed” if none fledged. The number of occupied sites with known breeding success and for which habitat and the age of the breeders were known varied from 79 to 180 per year for males (total 2,956 attempts) and from 30 to 147 per year for females (total 2,181 attempts). Breeding success was known for 91% of these sites, and this uncertainty did not vary with habitat type or the breeder's age (Wilcoxon signed rank tests; all *p* values > 0.25). Furthermore, the lack of a covariation between annual estimates of breeding success and proportion of nests with unknown fate (Spearman's rank correlation rho = −0.09, *p* value = 0.69) suggests that breeding success uncertainty induced no bias in our estimates of breeding success.

#### 2.2.2 Fledglings

Data on the number of fledglings produced came from the successful attempts. We included data from all occupied sites with records of the number of fledglings in the total study area. We defined the number of fledged Young as the number of chicks ringed when 4–8 days Old, minus the number of chicks found dead in the nest after fledging. The number of monitored sites for which the number of fledglings was known and for which habitat and age of the breeder were known varied from 31 to 105 per year for males (total 1,174) and from 25 to 87 per year for females (total 956). Compared to breeding success, sample size is lower as some territory sites were successful but their exact number of fledglings produced was unknown; this uncertainty was not related to habitat type or the age of the breeders or varying with the number of fledglings (all *p* values >0.14). Sample size is lower for females than males because it is more difficult to determine the age of unmarked females entering the breeding population than it is for males.

#### 2.2.3 First‐year recruitment

First‐year local breeding recruitment (probability to survive from successful fledgling to breeding as one year Old and stay in the study population) and transition probabilities between habitat types Short and Tall (from a natal habitat type to the other habitat type as breeder) were estimated for fledglings marked with an individual combination of color‐rings from successful nests in the central area between 1993 and 2015 (*N* = 4,993) that were resighted in the central area as breeders in the subsequent year (between 1994 and 2016). Juvenile wheatears cannot be sexed based on plumage during their first summer. Yet, estimation of sex‐specific parameters seemed relevant because more males than females were resighted as breeders the subsequent year (296 males, 235 females) and survival, movements between sites and resighting probabilities are known to vary between sexes of adult breeders (Arlt & Pärt 2007, 2008; Low et al., 2010). To estimate sex‐specific parameters, we assumed an even sex ratio among ringed nestlings and randomly attributed a sex to the nestlings that were never resighted (2,200 males, 2,262 females) so that the sex ratio of all ringed nestlings was even (2,496 males, 2,497 females).

#### 2.2.4 Adult apparent survival

Adult apparent survival (probability to survive and stay in the study population) and transition probabilities between breeding habitat types (from a breeding habitat type to the other) were estimated for breeders marked with an individual combination of color‐rings and for which we knew age‐class, habitat type, and breeding success at first capture (first captured as adults in the central area between 1993 and 2015; 559 males and 621 females breeding on 154 different territories). Individuals were resighted as breeders in the central area from 1994 to 2016 during the monitored breeding seasons (Low et al., 2010; Pärt, Knape, Low, Öberg, & Arlt, 2017). Hence, we model the dynamics of the local population based on local apparent survival rather than attempting to estimate real survival rates.

#### 2.2.5 Population size

We modeled the studied breeding population in the central area (40 km^2^) for which we had more detailed demographic data and monitoring was consistent in all years. Northern wheatears are typically socially monogamous in our study population and cases of social polyandry or polygyny are extremely rare. Therefore, as population size, we used the number of sites occupied by ringed or unringed breeders. Because we used age‐class‐ and habitat‐specific data, we only included sites for which the occupancy status (occupied/vacant), the habitat type (Short or Tall) and, for males, the age‐class (one year Old or older) of the breeder was known every year from 1993 to 2016 (*N* = 87 territory sites) Because female age‐class is more difficult to determine, the use of age‐class‐specific data would have severely limited our sample sizes on age‐specific count data (Arlt et al., 2008). We therefore analyzed males and females separately and used female data irrespective of age for the female‐based IPM (*N* = 117 territory sites).

To estimate how much each habitat type added to the population, we calculated habitat‐specific productivity as

$$\text{Prod}=\left(\text{first‐year}\;\text{recruits}\;\text{from}\;\text{hab}\;H\;+\;\text{app}\;\text{surv}\;\text{of}\;\text{ad}\;\text{from}\;\text{hab}\;H\right)/\text{no.}\;\text{ad}\;\text{hab}\;H,$$

that is, the number of fledglings produced at sites of a given habitat at year *t* that recruited to the studied population at year *t* + 1 plus the number of breeders in the given habitat at year t apparently surviving and breeding in the study area the year *t* + 1 divided by the number of breeders in the given habitat at year *t* (Heinrichs, Lawler, & Schumaker, 2016).

### 2.3 Integrated population model

**Figure 1:**
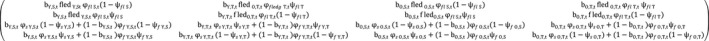
Transition matrix A_t_. Parameter **b** corresponds to breeding success, **fled** is the number of fledgling males from successful sites, φ is apparent survival, and Ψ are transition probabilities between the two habitats. Subscript ***fl*** refers to estimates for fledglings, and ***s* a**nd ***f*** refer to successful and failed breeders. Subscripts **Y** and **O** correspond to Young and Old male breeders, **S** and **T** correspond to habitats with Short and Tall ground vegetation, and **t** corresponds to time (year)

We combined the different sources of demographic information into an IPM. Integrating all available sources of information may improve precision of parameter estimates (Schaub & Abadi, 2011) and allows for the estimation of demographic parameters for which no explicit data are available, in our case apparent immigration (Abadi, Gimenez, Ullrich, Arlettaz, & Schaub, 2010). Our model describes a population of breeders in two different habitat types and hence with four categories of individuals: breeding on Short or Tall habitat type and one year Old (Young; Y) or older (Old; O). The model accounted for demographic stochasticity but a deterministic version can be described as:

NBY ShortNBY TallNBOShortNBOTallt+1=At×NBY ShortNBY TallNBOShortNBOTall+ImY ShortImY TallImOShortImOTallt+1

where **NB** is the number of breeders and **Im** is the number of apparent immigrants. Specifically, apparent immigrants are the residual number of individuals that are not predicted by the estimated local vital rates and population structure (the number of breeders in each age‐class and habitat type) as derived from the integration of data on vital rates and population count (Altwegg, Jenkins, & Abadi, 2014). The matrix $A_{t}$ (Figure 1) is the 4 × 4 projection matrix describing the transitions between each category of individuals from a year to the next (through fecundity, apparent survival, and transitions between habitat types; see details of the matrix in Supporting Information Appendix S1). Breeders’ apparent survival and transition probabilities from one habitat type to another were allowed to be conditional upon breeding success (Low et al., 2010; Pärt, Arlt, Doligez, Low, & Qvarnström, 2011). Therefore, breeding success can contribute to both the fecundity of the breeders but also their apparent survival and habitat transition probabilities. The female model did not account for age and therefore reduces to two categories of individuals (Short, Tall) using a 2 × 2 projection matrix. Because we have more complete age‐specific data for males, we primarily present results from the male‐based IPM (see also Arlt et al., 2008). Results from the female IPM were qualitatively similar from those of the male‐based model although not as distinct as for males and are presented in Supporting Information Appendix: Figure S1. We also estimated age‐specific female demographic parameters from breeding and capture resighting data in additional separate models out of the IPM (Table S1).

#### 2.3.1 Breeding success and number of fledglings

We use data on breeding success in a separate submodel than the number of fledglings and juvenile recruitment rate in order to explicitly model the relation between breeding success and other parameters like apparent survival and habitat transitions and because we model fledglings conditionally on successful breeding (Arlt & Pärt, 2007; Low et al., 2010). The number of successful sites counted each year per breeder's age‐class and habitat *B*_a,h,t_ was assumed to follow a binomial distribution with *B*_t_ ~ *B* (*b*_t_, *R*_t_) with *R*_t_ the number of monitored sites for which the breeding success was known. The number of fledglings from successful sites each year F_t_ minus the number of sites for which the number of fledglings is known S_t_ was assumed to be Poisson distributed so that *F*_t_−*S*_t_ ~Poisson (*S*_t_ (2fled_t_−1)) where 2fled_t_ is two times the number of fledglings of the modeled sex (assuming an even sex ratio). *F*_t_ was subtracted by *S*_t_ and 2fled_t_ by one since the restriction to successful breeding attempts implied at least one fledgling while a Poisson distribution is bounded from zero. We modeled the logit of breeding success and the log of the number of fledglings produced by successful broods with random year variation.

#### 2.3.2 Apparent survival and recruitment

Capture mark resighting data of adults were analyzed via a multi‐event model allowing for state uncertainty (Pradel, 2005). The state process describes apparent survival and transition probabilities of successful and failed breeders of the two age‐classes and habitat types. Transitions from states in year *t* to states in year *t* + 1 were set to follow a categorical distribution (see below for details of the transition matrix). The observation process links the true states and the observed states (i.e., events) via a categorical distribution accounting for imperfect detection and undetermined states (detection probability and state certainty set to be constant across years for each sex). Apparently alive but unobserved birds were assumed to be present in the study area by the model. Mark resighting data of fledglings were analyzed similarly but with two possible states in year t (born on Short or Tall sites) and only one resighting event in year *t* + 1 with two possible states for alive birds (Young breeding on Short or Tall habitat regardless of their breeding success) with again the possibility of unknown states. The detection probability of a Young breeder was assumed the same than for an Old breeder. We modeled the logit of recruitment and apparent survival rates with random year variation while transition probabilities were set to be constants through time.

#### 2.3.3 Count data

Population counts of Young and Old breeders in Short and Tall sites were analyzed using state space models with the above‐mentioned models describing the underlying population process. To account for demographic stochasticity, we modeled the numbers of locally produced and apparently immigrant breeding birds using Poisson and Binomial distributions (see Supporting Information Appendix S1 below for more details on the model). The numbers of apparent immigrants for each age‐class and habitat were estimated independently for each year (i.e., no constrained mean and variance across years). Because a few breeders may have been missed or double counted (e.g., if the same unringed breeders attempted to breed at two different territories within a season), we accounted for observation errors by linking counts of breeders (for males distinguishing Young and Old) in Short and Tall habitat to the underlying state process using Poisson distributions.

### 2.4 Model fitting

We constructed Bayesian IPMs inspired by examples in Kéry and Schaub (2011) and Weegman et al. (2016) using JAGS, version 4.2.0 (Plummer, 2015) run using the R2jags package (Su & Yajima, 2012) in Program R, version 3.3.2 (R Core Team, 2016). We estimated parameters using vague priors (normal distributions of mean 0 and precision 0.001 and uniform distributions between 0 and 10 for mean and variance parameters on the log and logit scales, uniform distributions between 0 and 1 for resighting and state certainty probabilities, uniform distribution between −5 and 20 for the expected yearly number of immigrants in each age‐class and habitats, normal distribution with count on year 1 as mean and precision of 0.01 for each expected age‐ and habitat‐specific numbers of occupied sites on year 1). Posterior samples from three Markov Chain Monte Carlo (MCMC) chains were based on 30,000 iterations after a burn‐in of 10,000 and thinning interval of 3 for each IPM (see Supporting Information Appendix S4). Model convergence was confirmed both visually and by using the “R hat” Gelman–Rubin statistic (Gelman & Rubin, 1992). We assessed the goodness of fit of all our submodels using postpredictive checks and the R package R2ucare (Gimenez, Lebreton, Choquet, & Pradel, 2018) and found no clear evidence for lack of fit (Supporting Information Appendix S3).

In the results, we present the means [and 95% CIs] from the posterior distributions of interest.

### 2.5 Identifying the past and potential drivers of the realized population growth

Using the IPM, we estimate how the different habitat‐specific demographic parameters contribute to the realized population growth rate. First, we quantify the actual past contributions by estimating the average and year‐specific contributions of the habitat‐specific demographic parameters to the variation in the realized growth rate of the population. We do this using a transient life table response experiment (transient LTRE) where we calculated the overall past contribution of each parameter to the variance of the annual population growth following Koons, Iles, Schaub, and Caswell (2016) taking into account the potential covariation between the focal parameter and the other parameters. Unlike asymptotic LTRE, transient LTRE allows to estimate the contribution of population structure in terms of age and habitat, and the contribution of the apparent immigrants in addition to the demographic parameters. Because we were interested in the annual variation, we also calculated the year‐specific contributions of the parameters to annual variation in realized growth rate, that is, a parameter's change between year t and year *t* + 1 times its associated sensitivity at time t. In this calculation, the covariation between parameters cannot be accounted for. Details for all calculations are found in Supporting Information Appendix S2.

Second, we quantify the potential contributions by estimating how sensitive the realized growth rate would be to a given change of each parameter. We do this by estimating real‐time transient stochastic elasticities of the geometric population growth rate that are estimated directly from observed temporal variation (Haridas et al., 2009). Rather than assuming a model of stochastic change in vital rates, those real‐time elasticities are estimated directly from data. Here, we used variation over the whole study period, that is, our real‐time transient elasticities were based on accumulated changes in the geometric mean population growth rate to yearly small changes in each parameter calculated between the first and the last year of study. Details for calculations are found in Supporting Information Appendix S2.

Because we find some age differences in demographic parameters, we estimate the parameters’ contribution to the realized population growth rate using the habitat‐ and age‐specific IPM, and hence results refer to male‐specific data.

## 3 RESULTS

### 3.1 Habitat‐specific demographic estimates

The estimates of demographic parameters varied with habitat type, with point estimates of apparent survival and fecundity parameters being generally lower on Tall sites as compared to Short sites (see estimates in Table 1 for males and Table S1 for females). Thus, there was no evidence of compensation between vital rates with respect to habitat‐specific demography. Age‐specific breeding success, number of fledglings, and fledging recruitment had a posterior probability of over 95% of being higher on Short sites than on Tall sites, suggesting clear differences. For all apparent survival parameters of breeders, this posterior probability was less than 95%, suggesting less clear differences. In addition, the temporal variation for some demographic parameters differed between habitat types (Table 1). For example, the estimated breeding success of Old breeders was lower and more variable in Tall sites than in Short sites (Table 1) while recruitment rate of fledglings born in Tall sites was lower and on average less variable than on Short sites (Table 1). Although apparent immigration rate (i.e., as estimated by the residual in the IPM) also varied among years, there were no clear differences between habitat types (Table 1) and immigration rates averaged 0.44 [95% CI: 0.39–0.49] across years.

**Table 1:** Estimated arithmetic means and coefficient of temporal variance (for parameters with temporal variation) of demographic parameters for males with associated 95% credible intervals

Parameter	Mean [95% BCI]	CV [95% BCI]
**n**_Y,Short_	0.20 [0.18–0.23]	0.38 [0.29–0.47]
**n**_O,Short_	0.41 [0.38–0.43]	0.21 [0.16–0.26]
**n**_Y,Tall_	0.16 [0.14–0.17]	0.41 [0.31–0.51]
**n**_O,Tall_	0.23 [0.21–0.25]	0.34 [0.26–0.42]
**b**_Y,Short_	0.71 [0.68–0.75]	0.09 [0.02–0.16]
**b**_O,Short_	0.81 [0.79–0.83]	0.07 [0.04–0.10]
**b**_Y,Tall_	0.60 [0.55–0.65]	0.12 [0.01–0.23]
**b**_O,Tall_	0.66 [0.63–0.70]	0.17 [0.11–0.23]
**f**_Y,Short_	2.58 [2.46–2.72]	0.05 [0.00–0.11]
**f**_O,Short_	2.82 [2.73–2.91]	0.02 [0.00–0.06]
**f**_Y,Tall_	2.32 [2.17–2.48]	0.07 [0.01–0.15]
**f**_O,Tall_	2.47 [2.34–2.60]	0.04 [0.00–0.11]
**φ** *_fledg_*_Short_	0.09 [0.08–0.10]	0.46 [0.22–0.68]
**φ** *_fledg_*_Tall_	0.06 [0.04–0.08]	0.25 [0.01–0.60]
**φ** *_success_*_Y,Short_	0.46 [0.39–0.53]	0.13 [0.01–0.32]
**φ** *_success_*_O,Short_	0.55 [0.50–0.60]	0.11 [0.00–0.23]
**φ** *_success_*_Y,Tall_	0.40 [0.30–0.50]	0.15 [0.01–0.41]
**φ** *_success_*_O,Tall_	0.40 [0.30–0.50]	0.16 [0.01–0.42]
**φ** *_fail_*_Y,Short_	0.49 [0.35–0.63]	0.28 [0.01–0.63]
**φ** *_fail_*_O,Short_	0.31 [0.21–0.41]	0.37 [0.12–0.82]
**φ** *_fail_*_Y,Tall_	0.37 [0.24–0.51]	0.40 [0.01–0.91]
**φ** *_fail_*_O,Tall_	0.30 [0.19–0.43]	0.32 [0.01–0.80]
**Ω**_Y, Short_	0.14 [0.11–0.17]	0.72 [0.53–0.95]
**Ω**_O, Short_	0.11 [0.08–0.14]	0.86 [0.62–1.16]
**Ω**_Y, Tall_	0.09 [0.07–0.12]	0.82 [0.60–1.10]

Parameter	Mean [95% BCI]	
**ψ** *_fledg_*_Short_	0.44 [0.36–0.52]	
**ψ** *_fledg_*_Tall_	0.54 [0.40–0.68]	
**ψ** *_success_*_Y__Short_	0.29 [0.19–0.39]	
**ψ** *_success_*_O__Short_	0.18 [0.13–0.23]	
**ψ** *_success_*_Y__Tall_	0.46 [0.30–0.62]	
**ψ** *_success_*_O__Tall_	0.45 [0.33–0.58]	
**ψ** *_fail_*_Y__Short_	0.39 [0.21–0.59]	
**ψ** *_fail_*_O__Short_	0.17 [0.05–0.33]	
**ψ** *_fail_*_Y__Tall_	0.51 [0.29–0.74]	
**ψ** *_fail_*_O__Tall_	0.71 [0.47–0.91]	
**p**	0.97 [0.94–0.99]	
**c**	0.88 [0.85–0.91]	
**c** *_f_*	0.97 [0.94–0.99]	

Note

**n** refers to the proportion of each age and habitat type among the occupied sites, **b** refers to breeding success, **f** to number of fledglings at successful sites, **φ** to apparent survival probabilities, **Ω** to the rate of apparent immigrant breeders relative to the total population, **ψ** to transition probabilities, **p** to resighting probability, and **c** to state certainties. Y and O refer to Young and Old males, and Short and Tall refer to sites with Short or Tall ground vegetation. All parameters were estimated from the integrated population model. See Table S1 for estimates for females.

**Figure 2:**
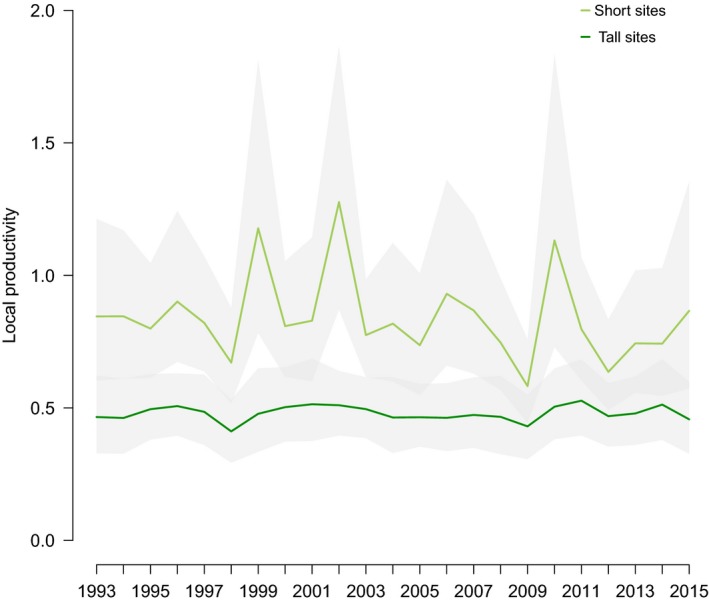
Productivity of Short sites (light green) and Tall sites (dark green) across years (means and 95% CIs). See main text for calculations

Productivity at Short sites was markedly higher than at Tall sites (geometric means 0.82 [0.75–0.89] and 0.48 [0.42–0.54], respectively) and on average more variable (arithmetic variance coefficients 0.27 [0.16–0.41] and 0.14 [0.06–0.23], respectively, Figure 2) although CI of their difference overlapped zero (0.12, [−0.016–0.28]).

### 3.2 Past drivers of realized population growth

#### 3.2.1 Average contributions to mean population growth rate variation

**Figure 3:**
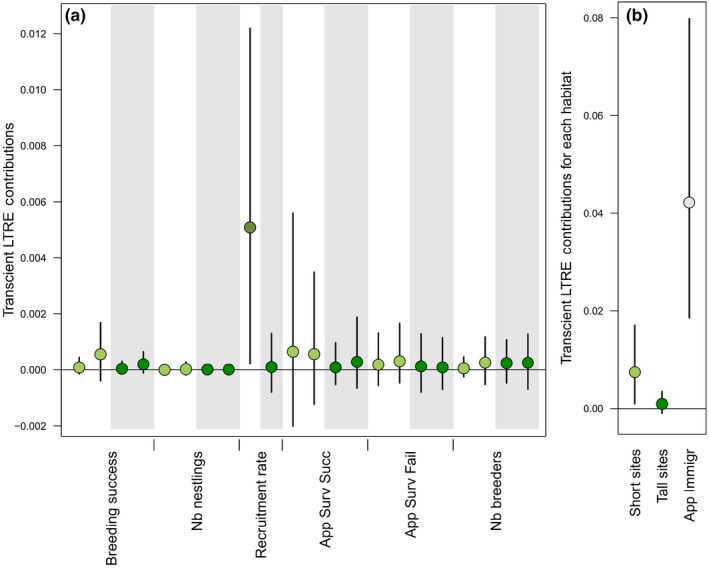
Transient LTRE contributions represent the part of variation in growth rate explained by the variation of each parameter (mean and 95% credible intervals for the male‐based model) **(a)**. Light green (white background) for Short sites and dark green (gray background) for Tall sites. For each parameter and habitat, values on the left are for Young breeders and on the right for Old breeders. Contributions can be summed to obtain, for example, the overall contribution of Short habitats (light green), Tall habitats (dark green), and apparent immigration (gray)

Transient LTRE contributions aim to estimate how much of the past observed annual variation in growth rate is explained by the variation of each parameter. Many demographic rates showed (at the most) only marginal contribution to realized population growth rate (Figure 3a). Overall, variation in the local demography (i.e., vital rates and age‐ and habitat‐specific population structure) explained on average 18% of the variation in realized growth rate, the remaining 82% of the variation being explained by the residual contribution of the apparent immigrants (but note the wide credible intervals; Figure 3b). Looking more specifically into the part of the variation in realized growth rate that was explained by local demography, this showed that most of the variation was explained by demographic rates in Short habitats (on average 84%), with this largely driven by the contribution of fledgling recruitment from Short sites (55%; Figure 3a). Population structure contributed little (on average 9%) to the variation explained by local demography (Figure 3a). Hence, looking at local demography, we found a clear contribution of Short habitat, and more specifically of the recruitment of fledglings from Short habitats, while we found no credible evidence for a contribution of Tall habitats (Figure 3).

#### 3.2.2 Year‐specific contributions and trends

**Figure 4:**
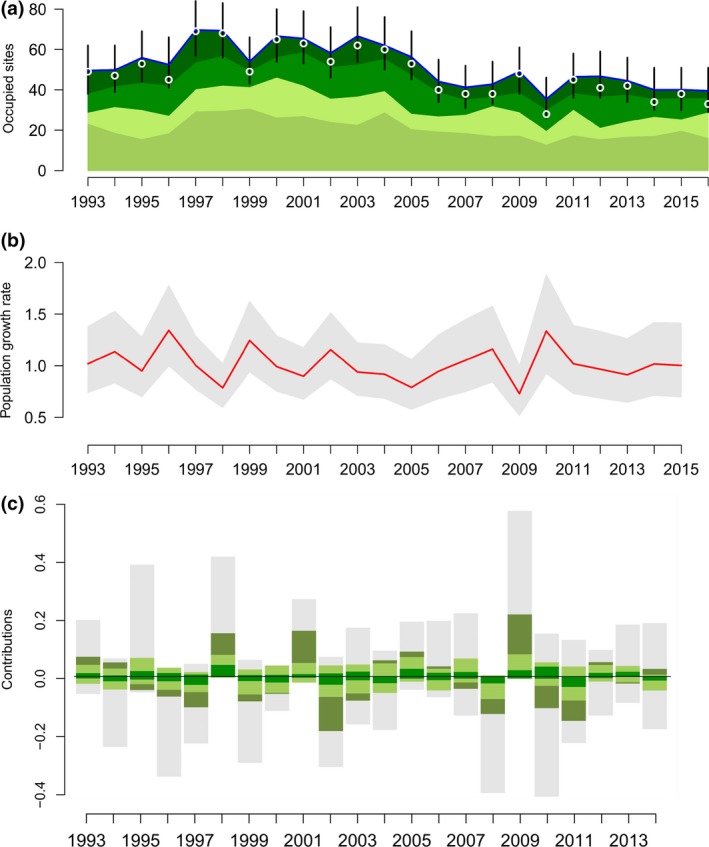
(a) Number of occupied sites (population size) by individuals in two age‐classes and in the two habitat types. The blue line (top) represents the estimated total population counts (and 95% CI), and white points the actual counts. Colors from the bottom to top show estimated counts for Short sites occupied by Old breeders, Short sites occupied by Young breeders, Tall sites occupied by Old breeders, and Tall sites occupied by Young breeders. **(b)** Annual population growth rate and 95% CI. **(c)** Average annual contributions to the change in population growth rate from recruitment of fledglings from Short habitat (olive green), the other demographic parameters on Short sites (combined in light green), Tall sites (combined in dark green; all green segments together show contributions from local demography), and the apparent immigration (for each age and habitat combined in gray). See Figure S1 for details on the average contributions of each habitat‐ and age‐specific parameter. Note that a change in population size in (a) (e.g., increased population in 1997) relates to a change in population growth rate in (b) observed the previous year and the contributions to this change in (c). For example, the population growth rate increased strongly from 1995 to 1996 with the largest contribution stemming from apparent immigration (1995) leading to the observed increase in population size in 1997

Population size varied substantially during the 24 years (Figure 4a,b). While the overall population did not show any general temporal trend (geometric mean growth rate: 0.99 [0.97–1.01]), there was some indication for a decline in the number of occupied Tall sites (0.97 [0.94–1.00]) but not of the number of Short sites (1.00 [0.98–1.02]). More specifically, the number of Young breeders decreased on Tall habitats but not on Short habitats (0.95 [0.89–0.99] and 1.04 [1.00–1.08], respectively). Meanwhile, there was no clear trend regarding Old breeders (on Tall and Short sites, respectively: 0.99 [0.95–1.03] and 0.98 [0.96–1.01]). Estimated population size tended to be slightly higher than the count data (Figure 4a), likely because of imperfect (albeit high) detection and/or occurrences of multiple pairs sharing a site in some years. There was temporal variation in the year‐specific contributions, although there was no obvious directional trend over time in the contribution of the two habitat types and their respective parameters (Figure 4c).

Although changes in population size seem mostly driven by changes in the proportion of apparent immigrants rather than by the local demography (Figure 3 and Figure 4c), the yearly contribution of apparent immigration showed wide 95% CI, always overlapping zero. On the other hand, the local demography may have contributed to the two “rebounding” events observed in 2000 and 2011 after drops in population size as indicated by its relative larger overall contribution to change in growth rate between 1998–1999 and 2009–2010 of 0.15 [0.02–0.29] and 0.21 [0.04–0.41], respectively, (Figure 4c) whose 95% CI did not overlap zero.

### 3.3 Potential drivers of realized population growth

**Figure 5:**
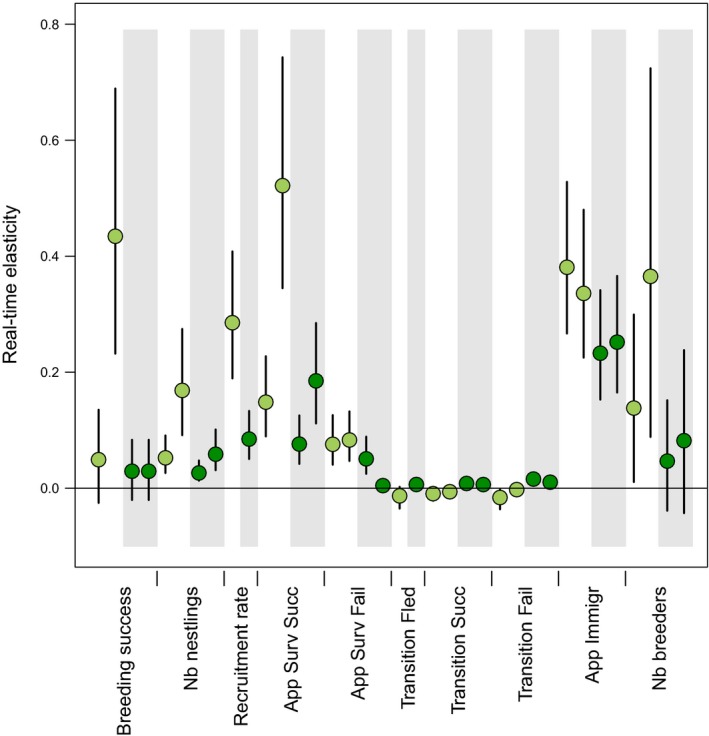
Real‐time elasticities (means and 95% credible intervals) estimated over the study period as how much a yearly small change in each parameter (0.001) would affect the geometric mean growth rate between 1993 and 2016. On light green (white background) for Short sites and dark green (gray background) for Tall sites. For each parameter and habitat, values on the left are for Young breeders and on the right for Old breeders

Elasticities aim to evaluate the potential influence of a given standardized change in each parameter on the population growth. The estimated real‐time elasticities suggest that the realized growth rate would be most sensitive to changes in demographic parameters of Short sites (Figure 5). More specifically, apparent survival of successful Old male breeders on Short sites and breeding success of Old male breeders on Short sites are associated with the highest elasticities (Figure 4). Furthermore, the number of male breeders in Short sites was also associated with high elasticities and especially so for Old males (Figure 5). Recruitment of male fledglings from Short sites and apparent immigration (with slightly higher values for Short sites) also present elasticity values >0.20 (Figure 5). Thus, changes in demographic parameters and abundances on Short sites should have stronger effects on the population growth rate than changes in demographic rates and abundances on Tall sites.

## 4 DISCUSSION

Like many other farmland bird species, the northern wheatear has markedly declined in abundance in agricultural landscapes. In Sweden, these declines have been most marked in the late 1970 s and 1980 s (61% decline between 1976 and 2000; Wretenberg, Lindström, Svensson, & Pärt, 2007). At the same time, agricultural landscapes have changed dramatically in terms of land use (e.g., a 10% reduction in areas of arable land and 22% reduction in areas of seminatural pastures between 1970 and 2000; Wretenberg et al., 2007). There seems to be a clear correlation, but quantifying the importance of different habitats for population growth is key for a better understanding of how land use changes affect population dynamics and could give incentives for how to reverse ongoing large‐scale population declines.

We were able to quantify the importance of different habitat types (here land use categories) and their respective utilization (habitat‐specific abundances of breeding birds) and vital rates for variation in growth rate of the whole population. The model used here can be applied to any population experiencing contrasting land uses or habitat types (e.g., organic vs. conventional, rural vs. urban areas, coniferous vs. deciduous woodland) providing valuable information that can be used to prioritize management actions, including identifying priority locations for management interventions (Zipkin & Saunders, 2018). In addition, by taking into account apparent immigration in our modeling, we could estimate the relative importance of local and habitat‐specific demography (at the study population scale) in contributing to local population growth; such estimates help identify the most relevant spatial scale for effective conservation (Baillie, Sutherland, Freeman, Gregory, & Paradis, 2000; Zipkin & Saunders, 2018). Below we discuss habitat‐specific demography, the importance and meaning of apparent immigration, the use of real‐time elasticities for identifying future potential interventions to benefit farmland birds and how such information may be applied when developing cost‐effective interventions to change population trends.

### 4.1 Habitat‐dependent demographic parameters and their temporal variation

In accordance with previous studies (Arlt et al., 2008; Low et al., 2010; Tye, 1992), we found that all estimated demographic parameters were higher on Short compared with Tall habitat sites; thus, demonstrating no demographic compensation is occurring in this system. Despite the consistent long‐term difference between the habitats in productivity, the potential contribution to local population size varied over time with Short sites being more variable than Tall sites (Figure 2). In our study, the greatest differences in productivity between the two habitats were observed in 1999, 2002, and 2010 (Figure 2) corresponding to three “rebounding” events following sudden decreases in population size (Figure 4a,b). In addition, the transient LTRE analysis of past variation in growth rate showed the importance of Short habitat for the dynamics of the population. Although credible intervals were wide, our results suggest the effect of Short sites to changes in population growth rate was mostly due to the contribution of recruitment of fledglings born in Short sites (representing 55% of the contribution of the local demography on growth rates, Figure 3a).

Contribution from Short sites in general, and from the recruitment of fledglings in particular, appeared consistent during the strongest shifts in population growth of the past 24 years (Figure 4c). This differs from a recent study on Lesser Scaups *Aythya affinis*, which found temporal shifts in parameters’ contributions (Koons et al., 2017). While no other IPM study has investigated temporal variability in contributions of habitat types, some studies suggest that even if some habitats are generally more favorable than others, this pattern may change in some years due to, for example, extreme weather conditions (Kindvall, 1995). However, we found no evidence for Tall habitats having more importance in extreme years to buffer variability in annual growth rates (cf. positive effects of habitat heterogeneity on population stability; Oliver, Roy, Hill, Brereton, & Thomas, 2010). Short habitats were the most important sites for population growth rates in all years.

### 4.2 Apparent immigration rate

Most population studies are based on data from populations open to immigration (and emigration) and where local immigration rates naturally contribute to total population growth rates. In our case, we estimated apparent immigration to have accounted for 44% of the central study population. Apparent immigration was also found to be high, although on average lower, in three more isolated, populations of Northern Wheatears in the Netherlands and may be an important determinant of their differences in growth rates (Oosten et al., 2015). Also, other studies of open populations suggest that immigration rates may be substantial (Schaub et al., 2012; Schaub, Jakober, & Stauber, 2013; Weegman, Arnold, Dawson, Winkler, & Clark, 2017). Moreover, in agreement with these other studies our study showed that the contribution of apparent immigration to total population growth rate was very large. Besides a high number of immigrants, there are two other possible reasons for these large contributions. First, all modeled demographic parameters are constrained around a mean with either random temporal variation or fixed as constant through time. Therefore, any overdispersed variation in a demographic parameter (e.g., exceptionally high survival) will not be fully estimated and will then contribute to the number of apparent immigrants (i.e., residual variation) for which no data are explicitly available, and particularly so in our case since these numbers were not constrained in our model. Second, population size, breeding parameters, apparent survival, and habitat transitions were estimated based from slightly different subsamples of the population. For example, CMR data were based on ringed birds only whereas population size was based on both ringed and unringed birds but only at sites for which we had information of habitat type and, for the male model of the age of the male breeder, in every year. The dynamics in these subsamples may slightly vary which would result in variation of apparent immigration. Since we do not know the origin (immigration status) of unringed individuals in our population, we have no explicit data on immigration that could be included in our model. Hence, without additional information to more specifically estimate dispersal (emigration and immigration), the estimated apparent immigration is a residual, estimating the discrepancy from shared parameters among different model components (Stenglein, Zhu, Clayton, & Van Deelen, 2015). These individuals not explained by the estimated local demography are typically seen as immigrants and usually constrained to some assumed mean value, making the assumption that their number or rate vary around a mean with random temporal variation (e.g., Schaub et al., 2013; Rushing et al., 2017; but see Altwegg et al., 2014). Applying such constraints on these parameters would have resulted in a (perhaps artificially) higher contribution of the local demography and an increased precision in the estimation of the parameters and their contribution (data not shown). Without such constraints, our model is conservative and provides the minimum contribution of the local demography and we call for caution in the interpretation of temporal contribution of apparent immigration when no explicit data are available.

### 4.3 Potential drivers of population growth and conservation implications

While LTRE analysis gives information on the actual variation of the parameters, real‐time elasticity analysis has the potential to investigate future scenarios of demographic changes. Therefore, combining both approaches provides a comprehensive overview of how feasible and efficient different management decisions may be (Manlik, Lacy, & Sherwin, 2018). In line with the LTRE analyses, we found that demographic parameters linked to Short habitats had a stronger potential to influence population growth rate than those of Tall habitats. In particular, recruitment rate of fledglings from Short habitat but also breeding success of Old breeders and apparent survival of successful Old breeders in Short sites had high elasticities and hence greatest potential to change the realized growth rate (Figure 5). High elasticities of apparent survival of Old breeders are in accordance with a previous study (Arlt et al., 2008), and the high value associated with breeding success is partly due to differences in apparent survival between successful and unsuccessful breeders (Low et al., 2010). This result suggests that reducing mortality of Old breeders on Short sites during breeding and increasing their breeding success would have a strong positive impact on population growth.

By investigating elasticities related to population structure (here in terms of age and habitat structure), we also estimated the sensitivity of the growth rate to the number of high‐ and low‐quality habitats occupied. We found that an increase of the number of Short sites occupied by Old breeders could have a distinct positive impact on population growth (Figure 5). We examined this by using real‐time elasticities analysis, which provides a flexible framework that can be used to test the effect of different management scenarios. For example as an illustration, we find that changing the proportion of Short sites occupied from 61% [59–64] to 68% [65–70] by adding Short sites occupied by Old males every year during the past 24 years would lead to a geometric mean growth rate of 1.08 [1.05–1.11] compared to the current geometric mean growth rate of 0.99 [0.97–1.01]. Habitat‐specific occupancy may be particularly realistic to manipulate compared to other demographic traits (Baxter, McCarthy, Possingham, Menkhorst, & McLean, 2006). Therefore, elasticity analysis on habitat‐specific occupancy may provide very useful information for landscape management and conservation decisions. High‐quality Short habitats such as grazed pastures have been declining in southern Sweden, most likely contributing to the observed national population declines. Hence, halting the decline of such habitats and converting low‐quality (e.g., ungrazed pasture, ruderal habitat) to high‐quality habitats would benefit the population. Taken together—because there was no evidence for compensation between demographic rates in different habitats—conservation measures should be focused on increasing the quality and abundance of land uses classified as Short habitats.

## CONFLICT OF INTEREST

None declared.

## AUTHORS CONTRIBUTIONS

M.P., D.A., T.P., J.K., M.L., and P.F conceived the study. T.P., D.A., J.K., M.L, and M.P. contributed to data collection. M.P. analyzed the data. M.P. led the writing of the manuscript with T.P. and D.A. All authors contributed critically to the drafts and gave final approval for submission.

## Data Availability

Data is available via the Dryad Digital Repository https://doi.org/10.5061/dryad.qh4fd46.
