# Supplementary material for: Quantifying the links between land use and population growth rate in a declining farmland bird
Source: Ecol Evol. 2019 Feb 5;9(2):868–79. doi: 10.1002/ece3.4766 (PMC6362438; doi:10.1002/ece3.4766)
Supplement: Supplementary file 1 [file ECE3-9-868-s001.docx]

**Appendix section summary :**

**Appendix S1:** Model description

**Appendix S2:** Identifying the realised and potential drivers of variation in growth rate and variance decomposition.

**Appendix S3:** Post predictive checks of the model

**Table S1:** Estimated demographic parameters for females

**Figure S1:** Transient LTRE contributions from the female-based IPM

**Figure S2:** Average annual contributions of each habitat- and age-specific parameter to the change in population growth rate.

**Appendix S4:** Jags code to fit the integrated population model (including code for post predictive checking).

**Appendix 1: Model description**

***LEGENDS:***

***h*** *is the habitat type: h1 or h2*

***a*** *is the age: Y (1 year old) or O (2+ years old)*

**φ** “apparent survival” defined as local breeding recruitment rate parameter (probability to survive, stay in the population and breed)

**ψ** transition parameter from habitat *h* to the other

Subscript **s** is for successful breeders and **f** for failed breeders

**b** breeding success

**fled** number of fledgling males produced per successful breeding attempts

**φ*_fledg_*** post fledgling local breeding recruitment rate parameter (probability to survive, stay in the population and breed the next year)

**ψ*_fledg´_*** post fledgling transition parameter from habitat *h* to the other

**p** re-sighting probability of a breeder

**c** state certainty. Probability to know the breeding success and the habitat of a re-sighted breeder.

**c*_f_*** Probability to know the habitat of a fledgling re-sighted as Young breeder.

**POPULATION COUNTS state space model**

**Observation process:**

C _a,h,t : observed number of sites occupied by a male of age a in habitat h at time t_

C _a,h,t_~Poisson(N *_a_*_,h,t_)

**System process:**

**Older males:**

N **_O,h1_**_,t_ = N *_previously failed_* **_Y,h1_**_,t-1_ **–** N *_previously failed_* **_Y_** *_moving from_* **_h1,_**_t-1_ + N *_previously failed_* **_O,h1_**_,t-1_ – N *_previously failed_* **_O_** *_moving from_* **_h1_**_,t-1_ + N *_previously failed_* **_Y_** *_moving from_* **_h2_**_,t-1_ + N *_previously failed_* **_O_** *_moving from_* **_h2_**_,t-1_ + N *_previously successful_* **_Y,h1_**_,t-1_ **-** N *_previously successful_* **_Y_** *_moving from_* **_h1_**_,t-1_ + N *_previously successful_* **_O,h1_**_,t-1_ – N *_previously successful_* **_O_** *_moving from_* **_h1_**_,t-1_ + N *_previously successful_* **_Y_** *_moving from_* **_h2_**_,t-1_ + N *_previously successful_* **_O_** *_moving from_* **_h2_**_,t-1_ + N_R_ **_O_**_,_**_h1_**_, t_

N **_O,h2_**_,t_ = N *_previously failed_* **_Y,h2_**_,t-1_ **–** N *_previously failed_* **_Y_** *_moving from_* **_h2,_**_t-1_ + N *_previously failed_* **_O,h2_**_,t-1_ – N *_previously failed_* **_O_** *_moving from_* **_h2_**_,t-1_ + N *_previously failed_* **_Y_** *_moving from_* **_h1_**_,t-1_ + N *_previously failed_* **_O_** *_moving from_* **_h1_**_,t-1_ + N *_previously successful_* **_Y,h2_**_,t-1_ **-** N *_previously successful_* **_Y_** *_moving from_* **_h2_**_,t-1_ + N *_previously successful_* **_O,h2_**_,t-1_ – N *_previously successful_* **_O_** *_moving from_* **_h2_**_,t-1_ + N *_previously successful_* **_Y_** *_moving from_* **_h1_**_,t-1_ + N *_previously successful_* **_O_** *_moving from_* **_h1_**_,t-1_ + N_R_ **_O_**_,_**_h2_**_, t_

N *_previously successful_* **_a_** *_moving from_* _h,t-1_~Binomial(ψ**_s_** _a,h,t-1_**,** N *_previously successful_* _a,h,t-1_)

N *_previously failed_* **_a_** *_moving from_* _h,t-1_~Binomial(ψ**_f_** _a,h,t-1_**,** N *_previously failed_* _a,h,t-1_)

N *_previously successful_* _a,h,t-1_~Binomial(φ**_s_** _a,h,t-1_**,** N *_successful_* _a,h,t-1_)

N *_previously failed_* _a,h,t-1_~Binomial (φ**_f_** _a,h,t-1_**,** N_a,h,t-1_-N *_successful_* _a_*_,_*_h,t-1_)

N *_successful_* _a,h,t-1_~Binomial(b _a,h,t-1_**,** N _a,h,t-1_)

N_R a,h,t_~Poisson(µ*_NR_* _a,h,t_)

**Young males:**

N **_Y,h1,_**_t_ = N*_fledg_* *_from_* **_Y,h1_**_,t-1_ **–** N*_fledg_* *_from_* **_Y_** *_moving from_* **_h1,_**_t-1_ + N*_fledg_* *_from_* **_O,h1_**_,t-1_ – N*_fledg_* *_from_* **_O_** *_moving from_* **_h1_**_,t-1_ + N*_fledg from_* **_Y_** *_moving from_* **_h2_**_,t-1_ + N*_fledg from_* **_O_** *_moving from_* **_h2_**_,t-1_ +N_R_ **_Y_**_,_**_h1_**_, t_

N **_Y,h2,_**_t_ = N*_fledg_* *_from_* **_Y,h2_**_,t-1_ **–** N*_fledg_* *_from_* **_Y_** *_moving from_* **_h2,_**_t-1_ + N*_fledg_* *_from_* **_O,h2_**_,t-1_ – N*_fledg_* *_from_* **_O_** *_moving from_* **_h2_**_,t-1_ + N*_fledg from_* **_Y_** *_moving from_* **_h1_**_,t-1_ + N*_fledg from_* **_O_** *_moving from_* **_h1_**_,t-1_ +N_R_ **_Y_**_,_**_h2_**_, t_

N *_fledg_* *_from_* **_a_** *_moving from_* _h,t-1_~Binomial(ψ**_fledg_** _h,t-1_**,** N*_fledg_* *_from_* _a,h,t-1_)

N *_fledg from_* _a,h,t-1_~Binomial(φ**_fledg_** _h,t-1_**,** N*_fledged_* _a,h,t-1_)

N*_fledged_* _a,h,t-1_~Binomial(0.5**,** Nmf*_fledged_* _a,h,t-1_)

Nmf*_fledged_* _a,h,t-1_- N *_successful_* _a,h,t-1_~Poisson(N *_successful_* _a,h,t-1_ × (2 × fled _a,h,t-1_-1))

**CAPTURE RECAPTURE DATA FOR ADULT BREEDERS:**

Multistate multi-event CR model

**z**_i,t_: True state of individual i at time t

$\mathbf{z}_{{i,f}_{i}}$ = $\mathbf{y}_{{i,f}_{i}}$= fs_i_ (state of individual i at first encounter)

**z**_i,t+1_|**z**_i,t_~Categorical($\Omega_{\boldsymbol{z}_{\boldsymbol{i}\boldsymbol{,}\boldsymbol{t}}\boldsymbol{,}\boldsymbol{1}\boldsymbol{\ldots}\boldsymbol{5}\boldsymbol{,}\boldsymbol{i}\boldsymbol{,}\boldsymbol{t}}$)

$\Omega_{\boldsymbol{z}_{\boldsymbol{i}\boldsymbol{,}\boldsymbol{t}}\boldsymbol{,}\boldsymbol{1}\boldsymbol{\ldots}\boldsymbol{4}\boldsymbol{,}\boldsymbol{i}\boldsymbol{,}\boldsymbol{t}}$= $\left( \begin{matrix} \varphi_{s a_{i},\mathbf{h1},t}{(1-\psi}_{s a_{i},\mathbf{h1},t})b_{\mathbf{O},\mathbf{h1},t+1} & \varphi_{s a_{i},\mathbf{h1},t}\psi_{s a_{i},\mathbf{h1},t}b_{\mathbf{O},\mathbf{h2},t+1} & \varphi_{s a_{i},\mathbf{h1},t}{(1-\psi}_{s a_{i},\mathbf{h1},t}){(1-b}_{\mathbf{O},\mathbf{h1},t+1}) & \varphi_{s a_{i},\mathbf{h1},t}\psi_{s a_{i},\mathbf{h1},t}{(1-b}_{\mathbf{O},\mathbf{h2},t+1}) & 1-\varphi_{s a_{i},\mathbf{h1},t} \\ \varphi_{s a_{i},\mathbf{h2},t}\psi_{s a_{i},\mathbf{h2},t}b_{\mathbf{O},\mathbf{h1},t+1} & \varphi_{s a_{i},\mathbf{h2},t}{(1-\psi}_{s a_{i},\mathbf{h2},t}{)b}_{\mathbf{O},\mathbf{h2},t+1} & \varphi_{s a_{i},\mathbf{h2},t}\psi_{s a_{i},\mathbf{h2},t}{(1-b}_{\mathbf{O},\mathbf{h1},t+1}) & \varphi_{s a_{i},\mathbf{h2},t}{(1-\psi}_{s a_{i},\mathbf{h2},t}{)(1-b}_{\mathbf{O},\mathbf{h2},t+1}) & 1-\varphi_{s a_{i},\mathbf{h2},t} \\ \varphi_{f a_{i},\mathbf{h1},t}{(1-\psi}_{f a_{i},\mathbf{h1},t})b_{\mathbf{O},\mathbf{h1},t+1} & \varphi_{f a_{i},\mathbf{h1},t}\psi_{f a_{i},\mathbf{h1},t}b_{\mathbf{O},\mathbf{h2},t+1} & \varphi_{f a_{i},\mathbf{h1},t}{(1-\psi}_{f a_{i},\mathbf{h1},t}){(1-b}_{\mathbf{O},\mathbf{h1},t+1}) & \varphi_{f a_{i},\mathbf{h1},t}\psi_{f a_{i},\mathbf{h1},t}{(1-b}_{\mathbf{O},\mathbf{h2},t+1}) & 1-\varphi_{f a_{i},\mathbf{h1},t} \\ \varphi_{f a_{i},\mathbf{h2},t}\psi_{f a_{i},\mathbf{h2},t}b_{\mathbf{O},\mathbf{h1},t+1} & \varphi_{f a_{i},\mathbf{h2},t}{(1-\psi}_{f a_{i},\mathbf{h2},t}{)b}_{\mathbf{O},\mathbf{h2},t+1} & \varphi_{f a_{i},\mathbf{h2},t}\psi_{f a_{i},\mathbf{h2},t}{(1-b}_{\mathbf{O},\mathbf{h1},t+1}) & \varphi_{f a_{i},\mathbf{h2},t}{(1-\psi}_{f a_{i},\mathbf{h2},t}{)(1-b}_{\mathbf{O},\mathbf{h2},t+1}) & 1-\varphi_{f a_{i},\mathbf{h2},t} \\ 0 & 0 & 0 & 0 & 1 \end{matrix} \right)$

States:

**1**: successful in FLH1

**2**: successful in FLH2

**3**: failed in FLH1

**4**: failed in FLH2

**5**: dead

**y**_i,t_: Observed state (i.e. event) of individual i at time t

**y**_i,t+1_|**z**_i,t+1_~Categorical($\theta_{\boldsymbol{z}_{\boldsymbol{i,t+1}}\boldsymbol{,1\ldots6, i,t}}$)

$\theta_{\boldsymbol{z}_{\boldsymbol{i,t+1}}\boldsymbol{,1\ldots6, i,t}}$= $\left( \begin{matrix} \mathrm{pc} & 0 & 0 & 0 & p(1-c) & 1-p \\ 0 & \mathrm{pc} & 0 & 0 & p(1-c) & 1-p \\ 0 & 0 & \mathrm{pc} & 0 & p(1-c) & 1-p \\ 0 & 0 & 0 & \mathrm{pc} & p(1-c) & 1-p \\ 0 & 0 & 0 & 0 & 0 & 1 \end{matrix} \right)$

**p** is recapture probability of a breeder and **c** is the probability to know the breeding status and the habitat of the breeder i.e. state certainty

Observed states (events):

**1**: seen successful in FLH1

2: seen successful in FLH2

3: seen failed in FLH1

4: seen failed in FLH2

5: seen but breeding success or habitat unknown

6: not seen

FLEDGLING CAPTURE RECAPTURE DATA:

Multistate multi-event CR model

**2**: seen successful in FLH

3: seen failed in FLH1

4: seen failed in FLH2

**5: seen but breeding success or habitat unknown**

6: not seen

**FLEDGLING CAPTURE RECAPTURE DATA:**

Multistate multi-event CR model

There is only one recapture event for each fledgling. At time f_i_+1

$\mathbf{z}_{\boldsymbol{fledg} i,t}$:True state of individual i at time t

$\mathbf{z}_{\boldsymbol{fled} {i,f}_{i}}g$ = $\mathbf{y}_{\boldsymbol{fledg} {i,f}_{i}}$= fs_i_ (state of individual i at first encounter)

$\mathbf{z}_{{\boldsymbol{fledg} i,f}_{i}+1}$ |$\mathbf{z}_{\boldsymbol{fledg} {i,f}_{i}}$~Categorical($\Omega_{fledg\boldsymbol{z}_{\boldsymbol{i}\boldsymbol{,}\boldsymbol{f}_{\boldsymbol{i}}}\boldsymbol{,}\boldsymbol{1}\boldsymbol{\ldots}\boldsymbol{3}\boldsymbol{,}\boldsymbol{i}\boldsymbol{,}\boldsymbol{f}_{\boldsymbol{i}}}$)

$\Omega_{fledg\boldsymbol{z}_{\boldsymbol{i}\boldsymbol{,}\boldsymbol{f}_{\boldsymbol{i}}}\boldsymbol{,}\boldsymbol{1}\boldsymbol{\ldots}\boldsymbol{3}\boldsymbol{,}\boldsymbol{i}\boldsymbol{,}\boldsymbol{f}_{\boldsymbol{i}}}$= $\left( \begin{matrix} \varphi_{fledg,\mathbf{h1},t}{(1-\psi}_{fledg,\mathbf{h1},t}) & \varphi_{fledg,\mathbf{h1},t}\psi_{fledg,\mathbf{h1},t} & 1-\varphi_{fledg,\mathbf{h1},t} \\ \varphi_{fledg,\mathbf{h2},t}\psi_{fledg,\mathbf{h2},t} & \varphi_{fledg,\mathbf{h2},t(1-}\psi_{fledg,\mathbf{h2},t}) & 1-\varphi_{fledg,\mathbf{h2},t} \end{matrix} \right)$

States:

**1**: in FLH1. At time f_i_ it means the habitat where the fledgling was raised and at f_i_+1 it means the habitat where it breeds

**2**: in FLH2

**3**: dead

$\mathbf{y}_{\boldsymbol{fledg} {i,f}_{i}+1}$: Observed state (i.e. event) of individual i at time f_i_+1

$\mathbf{y}_{{\boldsymbol{fledg} i,f}_{i}+1}$|$\mathbf{z}_{{\boldsymbol{fledg} i,f}_{i}+1}$~Categorical($\theta_{fledg\boldsymbol{z}_{\boldsymbol{i}\boldsymbol{,}\boldsymbol{f}_{\boldsymbol{i}}\boldsymbol{+}\boldsymbol{1}}\boldsymbol{,}\boldsymbol{1}\boldsymbol{\ldots}\boldsymbol{4}\boldsymbol{,}\boldsymbol{i}\boldsymbol{,}\boldsymbol{f}_{\boldsymbol{i}}}$)

$\theta_{fledg\boldsymbol{z}_{\boldsymbol{i}\boldsymbol{,}\boldsymbol{f}_{\boldsymbol{i}}\boldsymbol{+}\boldsymbol{1}}\boldsymbol{,}\boldsymbol{1}\boldsymbol{\ldots}\boldsymbol{4}\boldsymbol{,}\boldsymbol{i}\boldsymbol{,}\boldsymbol{f}_{\boldsymbol{i}}}$= $\left( \begin{matrix} pc_{f} & 0 & p{(1-c}_{f}) & 1-p \\ 0 & pc_{f} & 0 & 1-p \\ 0 & 0 & 0 & 1 \end{matrix} \right)$

$c_{f}$ is the probability to know the habitat of the breeder i.e. state certainty

Observed states (events):

**1**: seen in FLH1

**2**: seen in FLH2

**3**: **seen but habitat unknown**

**6**: not seen

**BREEDING SUCCESS DATA:**

**B**_a,h,t_ :The number of counted successful sites

**R**_a,h,t_ :The number of sites for which the breeding success is known

**B**_a,h,t_~Binomial(b_a,h,t_,R_a,h,t_)

**NUMBER OF FLEDGLINGS DATA:**

**F**_a,h,t_ :The number of counted fledglings (among successful sites of known number of fledglings)

**S**_a,h,t_ :The number of sites for which the number of fledglings is known

**F**_a,h,t_**-S**_a,h,t_ ~Poisson(**S**_a,h,t_ **(2fled**_a,h,t_-1))

**CONSTRAINS AND PRIORS**

For **fled**_a,h,t_ (mean number of fledged males for each parent’s age and habitat)

log(**fled**_a,h,t_)= mu_a,h_+ε_a,h,t_

mu_a,h_~Normal(0, 1e+06)

ε_a,h,t_~Normal(0,sigma)

sigma~Uniform(0,10)

For **µ*_NR_* _a,h,t_** (mean residual number of breeders for each age and habitat)

**µ*_NR_* _a,h,t_**~Uniform(-5,20)

For **φ*_f ledg_***_,h,t_ **φ*_f_*** _a,h,t_*,,* **φ*_s_*** _a,h,t,_ **b**_a,h,t_

logit(parameter)= mu_a,h_+ε_a,h,t_

mu_a,h_~Normal(0, 1e+06)

ε_a,h,t_~Normal(0,sigma)

sigma~Uniform(0,10)

For*,* **ψ*_f ledg_***_,h,t_ **ψ*_f_*** _a,h,t_*,* **ψ*_s_*** _a,h,t,_

logit(parameter)= mu_a,h_

mu_a,h_~Normal(0, 1e+06)

p~Uniform(0,1)

c~Uniform(0,1)

c_f_~Uniform(0,1)

**Initial values are available from the script file in Appendix 4.**

**Appendix 2: Identifying the realised and potential drivers of variation in growth rate and Variance decomposition**

*Identifying realised drivers*: *Transient life table response experiment*

We calculated the overall past contribution of each parameter to the temporal variance of the population growth following Koons et al. (2016) as:

$$\sum_{j} cov(\theta_{i,t}\theta_{j,t})\frac{\partial\lambda_{t}}{\partial\theta_{i,t}}\left. \frac{\partial\lambda_{t}}{\partial\theta_{j,t}} \right|_{\overset{¯}{\theta}}$$

Here $\left. \frac{\partial\lambda_{t}}{\partial\theta_{i,t}} \right|_{\overset{¯}{\theta}}$ is the sensitivity of the growth rate to change in the focal parameter θ_i_ at the mean population structure and mean values of the parameters (see below for details on the variance decomposition and the calculated solutions). The calculated contributions take into account the potential covariation between the focal parameter $\theta_{i}$ and the others $\theta_{j}$ that could exacerbate or nullify the effect of $\theta_{i}$ on the realised growth rate. In addition to the demographic parameters, we also considered the contribution of population structure (e.g. age and spatial (habitat) structure, *sensu* Koons et al. (2017), and the contribution of the apparent immigrants, defined as the number of apparent immigrants (for each of the four classes of breeders) at time t+1 divided by the total number of breeders at time t.

We also calculated the year specific contributions of the parameters to annual variation in realised growth rate (Koons et al., 2017) as:

($\theta_{i,t+1}-\theta_{i,t})\left. \frac{\partial\lambda_{t}}{\partial\theta_{i,t}} \right|_{\overset{¯}{\theta_{i}}}$

Hence, a parameter’s year specific contribution to growth rate of that time interval is its change between year t and year t+1 times its associated sensitivity at time t. Note that in this calculation, the covariation between parameters cannot be accounted for.

*Identifying potential drivers*: *real time elasticities*

To estimate how sensitive would the realised growth rate be to a given change of each parameter we calculated the “real time” sensitivities of the realised (geometric) growth rate calculated between the first (t=1) and the last year (t=T) of study to yearly small changes **∂** in each parameter ***θ***_i_. To do so, we built one population model (without demographic stochasticity) for each parameter of interest, including transition probabilities and abundances in each habitat and age class, and we applied a small increase (**∂**=0.001) to the focal parameter every year in order to estimate the perturbed growth rate$\lambda_{1-T}^{\theta_{i}+\partial}$. We also build the same model without perturbation to estimate $\lambda_{1-T}^{\theta}$ and all models were run simultaneously with our IPM (estimating the demographic parameters and abundances). We then calculated the sensitivity of the geometric growth rate (between year 1 and T) to each parameter ***θ_i_*** as $\frac{\lambda_{1-T}^{\theta_{i}+\partial}-\lambda_{1-T}^{\theta}}{\partial}$ and their associated elasticities as $\frac{\lambda_{1-T}^{\theta_{i}+\partial}-\lambda_{1-T}^{\theta}}{\partial}\times\frac{\bar{\theta}_{i}}{\lambda_{1-T}^{\theta}}$ to evaluate their relative potential influence on population growth, that is, how sensitive would the realised growth rate be to a given standardised change of the focal parameter. Posterior samples were obtained following the same settings as for our IPM.

*Variance decomposition*

We here give the details for the calculation of the contributions of the demographic parameters and population sizes to the variance of the growth rate. These are based on an approximation to the variance given in eq. 5 of Koons et al. (2016):

$$Var(\lambda_{realised,t})\approx\sum^{i} \sum^{j} Cov(\theta_{i,t},\theta_{j,t})\frac{\partial\lambda_{realised,t}}{\partial\theta_{i,t}}|_{\theta_{t}=\overline{\theta}}\frac{\partial\lambda_{realised,t}}{\partial\theta_{j,t}}|_{\theta_{t}=\overline{\theta}}$$

where $\theta_{\cdot,t}$ are parameters that determine the growth rate at time $t$, and $\overline{\theta}$ is the mean of the parameter over some time period.

The background for this equation is to view $\lambda_{realised,t}$ as a function of $\theta$, say $\lambda_{realised,t}=f(\theta_{t})$. Hence, the variation in $\lambda_{realised,t}$ over time is assumed to be a consequence of, and fully determined by, the variation in the parameters $\theta$. For now, we assume that the vector $\theta$ consists of two parts, $\theta=(\delta,\mathbf{n})'$, where $\delta$ is a vector of demographic parameters (survival, fecundity, breeding success etc) that determine the matrix $\mathbf{A}$ and $\mathbf{n}$ is a vector of the number of individuals in the different stages. We will later add a third part consisting of the number of immigrants.

The expression above is obtained from a first order Taylor approximation of $f$ around the mean of the parameters. For the Taylor approximation of $f$ Koons et al. use the fact that $f(\theta_{t})=f(\delta_{t},\mathbf{n}_{t})=f(\delta_{t},{\overset{̃}{\mathbf{n}}}_{t})$ where ${\overset{̃}{\mathbf{n}}}_{t}=\frac{\mathbf{n}_{t}}{||\mathbf{n}||}$ is the normalized population structure vector (i.e. the total population size does not affect the growth rate). They then use the Taylor expansion around the mean of the normalized vector ${\overset{̃}{\mathbf{n}}}_{t}$ instead of around the vector with numbers in stages. For $\theta_{t}$ that are close to the means, the Taylor approximation of $f$ is

$$f(\theta_{t})=f(\delta_{t},\mathbf{n}_{t})=f(\delta_{t},{\overset{̃}{\mathbf{n}}}_{t})\approx f(\overline{\delta},\overline{\overset{̃}{\mathbf{n}}})+\sum^{i} \frac{\partial f}{\partial\delta_{i,t}}|_{\theta=(\overline{\delta},\overline{\overset{̃}{\mathbf{n}}})}(\delta_{i,t}-\overline{\delta}_{i})+\sum^{i} \frac{\partial f}{\partial n_{i,t}}|_{\theta=(\overline{\delta},\overline{\overset{̃}{\mathbf{n}}})}({\overset{̃}{n}}_{i,t}-{\overline{\overset{̃}{n}}}_{i})$$

In the following we will use condensed notation containing only $\theta$, but it should be kept in mind that the derivatives should be evaluated at the mean of the normalized population structure.

To approximate the variance we need the following formula for decomposing the variance of a sum of stochastic variables $y_{i}$ with fixed (not stochastic) weights $a_{i}$:

$$Var(\sum^{i} a_{i}y_{i})=Cov(\sum^{i} a_{i}y_{i},\sum^{j} a_{j}y_{j})=\sum^{i} \sum^{j} a_{i}a_{j}Cov(y_{i},y_{j}).$$

Using this formula and replacing $f$ for its Taylor approximation we have

$$\begin{matrix} Var(\lambda_{realised,t}) & = & Var(f(\theta_{t}))\approx Var(f(\overline{\theta})+\sum^{i} \frac{\partial f}{\partial\theta_{i,t}}|_{\theta=\overline{\theta}}(\theta_{i,t}-\overline{\theta}_{i}))=Var(\sum^{i} \frac{\partial f}{\partial\theta_{i,t}}|_{\theta=\overline{\theta}}(\theta_{i,t}-\overline{\theta}_{i})) \\ & = & Cov(\sum^{i} \frac{\partial f}{\partial\theta_{i,t}}|_{\theta=\overline{\theta}}(\theta_{i,t}-\overline{\theta}_{i}),\sum^{j} \frac{\partial f}{\partial\theta_{j,t}}|_{\theta=\overline{\theta}}(\theta_{j,t}-\overline{\theta}_{j})) \\ & = & \sum^{i} \sum^{j} Cov(\frac{\partial f}{\partial\theta_{i,t}}|_{\theta=\overline{\theta}}(\theta_{i,t}-\overline{\theta}_{i}),\frac{\partial f}{\partial\theta_{j,t}}|_{\theta=\overline{\theta}}(\theta_{j,t}-\overline{\theta}_{j})) \end{matrix}$$

Now, since $\frac{\partial f}{\partial\theta_{i,t}}|_{\theta_{t}=\overline{\theta}}$ is evaluated at the mean of the parameters it is a constant and can be moved outside of the covariance giving

$$Var(\lambda_{realised,t})\approx\sum^{i} \sum^{j} \frac{\partial f}{\partial\theta_{i,t}}|_{\theta=\overline{\theta}}\frac{\partial f}{\partial\theta_{j,t}}|_{\theta=\overline{\theta}}Cov((\theta_{i,t}-\overline{\theta}_{i}),(\theta_{j,t}-\overline{\theta}_{j}))$$

A final simplification is that the terms $\overline{\theta}_{i}$ in the covariance can be deleted since they are constants:

$$Var(\lambda_{realised,t})\approx\sum^{i} \sum^{j} \frac{\partial f}{\partial\theta_{i,t}}|_{\theta=\overline{\theta}}\frac{\partial f}{\partial\theta_{j,t}}|_{\theta=\overline{\theta}}Cov(\theta_{i,t},\theta_{j,t})$$

This is the formula given in above and in Koons et al.

In applications, $Cov(\theta_{i,t},\theta_{j,t})$ has been estimated from the sample covariance between the parameters over the whole time period considered. This assumes that the covariance does not depend on time $t$ and results in a variance approximation that is also independent of time.

## Derivatives for population size

We here give a general formula for computing the derivatives with respect to population size, $\frac{\partial\lambda_{realised}}{\partial n_{i,t}}$ when there is no density dependence (i.e. the matrices $\mathbf{A}_{t}$ do not depend on $\mathbf{n}_{t}$). Computing the derivatives for the demographic parameters, $\frac{\partial f}{\partial\delta_{i,t}}$, proceeds as in Koons et al.

A general formula for the sensitivity of $\lambda_{realised,t}$ with respect to $n_{i,t}$ is

$$\frac{\partial\lambda_{realised,t}}{\partial n_{i,t}}=\frac{\partial}{\partial n_{i,t}}\frac{||\mathbf{A}_{t}\mathbf{n}_{t}||}{||\mathbf{n}_{t}||}=\frac{\sum^{j} \mathbf{A}_{t(j,i)}-\lambda_{realised,t}}{||\mathbf{n}_{t}||}=\frac{sum of column i\mathrm{of}\mathbf{A}_{t}-\lambda_{realised,t}}{||\mathbf{n}_{t}||}$$

Evaluated at the normalized population structure, this becomes

$$\frac{\partial\lambda_{realised,t}}{\partial n_{i,t}}|_{\mathbf{n}_{t}={\overset{̃}{\mathbf{n}}}_{t},\delta_{t}=\delta_{t}}=sum of column i\mathrm{of}\mathbf{A}_{t}-\lambda_{realised,t}$$

This is a general version of the expression for the sensitivities to population size given by Koons et al.

## Contributions from immigration

When there is immigration as in the analysis here, the growth rate becomes

$$\lambda_{realised,t}=\frac{||\mathbf{A}_{t}\mathbf{n}_{t}||+||N_{imm,t}||}{||\mathbf{n}_{t}||}$$

To decompose the variance in this case, we first reparametrize the immigration to ${\overset{^}{N}}_{imm,t}=\frac{N_{imm,t}}{||\mathbf{n}_{t}||}$ so that ${\overset{^}{N}}_{imm,t}$ is the immigration rate relative to the abundance in the previous year. With this definition the growth rates is

$$\lambda_{realised,t}=\frac{||\mathbf{A}_{t}\mathbf{n}_{t}||}{||\mathbf{n}_{t}||}+||{\overset{^}{N}}_{imm,t}||$$

Considering $\lambda_{realised,t}$ as a function of $(\delta_{t},\mathbf{n}_{t},{\overset{^}{N}}_{imm,t})$ the derivatives with respect to $\delta_{i,t}$ and $n_{i,t}$ remain unchanged and the derivatives with respect to ${\overset{^}{N}}_{imm,i,t}$ are 1. The derivatives and covariances then also need to be computed using ${\overset{^}{N}}_{imm,t}$.

An alternative might be to instead use the reparameterization ${\overset{˘}{N}}_{imm,t}=\frac{N_{imm,t}}{||\mathbf{A}_{t}\mathbf{n}_{t}||}$, i.e. standardized with respect to the final population size. Then

$$\lambda_{realised,t}=\frac{||\mathbf{A}_{t}\mathbf{n}_{t}||}{||\mathbf{n}_{t}||}(1+||{\overset{˘}{N}}_{imm,t}||)$$

Using this parameterization derivatives with respect to $\delta_{i,t}$ and $n_{i,t}$ computed without immigration need to be multiplied by $(1+||{\overset{˘}{N}}_{imm,t}||)$. The derivatives with respect to ${\overset{˘}{N}}_{imm,i,t}$ become equal to $\frac{||\mathbf{A}_{t}\mathbf{n}_{t}||}{||\mathbf{n}_{t}||}$.

**Solutions to the sensitivities of realized population growth rate to changes in** **vital rates.**

$$\lambda_{realised,t}=\frac{||\mathbf{A}_{t}\mathbf{n}_{t}||}{||\mathbf{n}_{t}||}+||{\overset{^}{N}}_{imm,t}||$$

For each age and habitat:

$\frac{{\partial\lambda}_{t}}{\partial f_{a,h,t}}$ *=* $b_{a,h,t}. \varphi_{fledg,h,t}\hat{n}_{a,h,t}$

$\frac{{\partial\lambda}_{t}}{\partial\varphi_{fledg,h,t}}$ *=*$b_{Y,h,t}f_{Y,h,t}. \hat{n}_{Y,h,t}+b_{O,h,t}f_{O,h,t}. \hat{n}_{O,h,t}$

$\frac{{\partial\lambda}_{t}}{\partial\varphi_{f a,h,t}}$ *=* ${(1-b}_{a,h,t}). \hat{n}_{a,h,t}$

$\frac{{\partial\lambda}_{t}}{\partial\varphi_{s a,h,t}}$ *=* $b_{O,h,t}. \hat{n}_{a,h,t}$

$\frac{{\partial\lambda}_{t}}{\partial b_{a,h,t}}$ *=* ${( f}_{a,h,t}. \varphi_{fledg,h,t} +\varphi_{s a,h,t}-\varphi_{f a,h,t})\hat{n}_{a,h,t}$

$\frac{{\partial\lambda}_{t}}{\partial{\hat{N}imm}_{a,h,t+1}}$ *=* 1

Where ${\overset{^}{N}}_{imm,t}$ is the proportion of residual individuals relative to the abundance in the previous year.

$\frac{{\partial\lambda}_{t}}{\partial\hat{n}_{a,h,t}}|_{\mathbf{n}_{t}={\overset{̃}{\mathbf{n}}}_{t},}$ *=* ${b_{a,h,t} f}_{a,h,t}. \varphi_{fledg,h,t} +b_{a,h,t}\varphi_{s a,h,t}+\left( 1-b_{a,h,t} \right)\varphi_{f a,h,t}-\left\| A_{t}\hat{n}_{t} \right\|$

**Appendix 3: Post predictive checks of the model**

The overall goodness of fit of our sub models for i) the **number of successful sites**, ii) the **number of fledglings among successful sites** and iii) **count data** were assessed using the χ² discrepancy metric (Gelman, Meng, & Stern, 1996). This metric compares the distance of the observed data to the model (discrepancy of data), calculated as:

$$D_{\chi^{2}}^{obs}= \sum_{h=1}^{2} \sum_{a=1}^{2} \sum_{t=1}^{24} \frac{{(y_{h,a,t}^{obs}-E\left( y_{h,a,t} | \theta\right))}^{2}}{Var\left( y_{h,a,t} | \theta\right)}$$

with the distance of “perfect” replicated data to the model (discrepancy of replicate data):

$$D_{\chi^{2}}^{rep}= \sum_{h=1}^{2} \sum_{a=1}^{2} \sum_{t=1}^{24} \frac{{(y_{h,a,t}^{rep}-E\left( y_{h,a,t} | \theta\right))}^{2}}{Var\left( y_{h,a,t} | \theta\right)}$$

where $y_{h,a,t}^{obs}$ and $y_{h,a,t}^{rep}$represent the observed or replicated data for each habitat, age class and year and θ represents the estimated subset of parameters.

Post predictive probabilities (reported as P in the figures below) inform on how many times in our posterior samples the distance of the replicated data to the expected mean is greater than the distance of the observed data to the expected mean. A value of 0.5 suggests a good fit while values close to 0 or 1 (classically below 0.05 or above 0.95) suggest a lack of fit. The slightly poorer fit observed for the number of fledglings and population size (habitat and age specific occupancy) is likely due to constrains associated with the use of Poisson distribution (variance=mean).


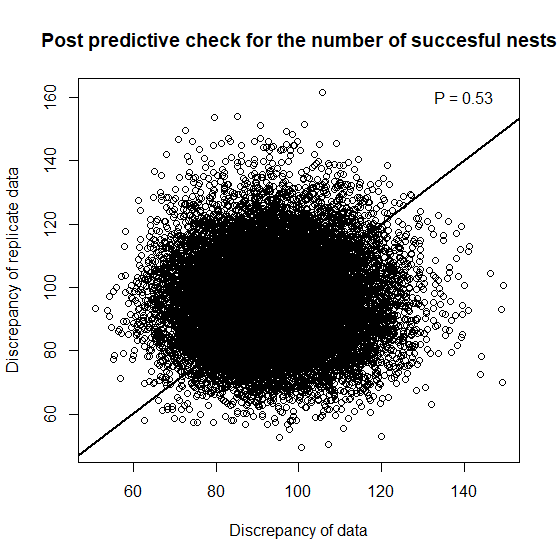


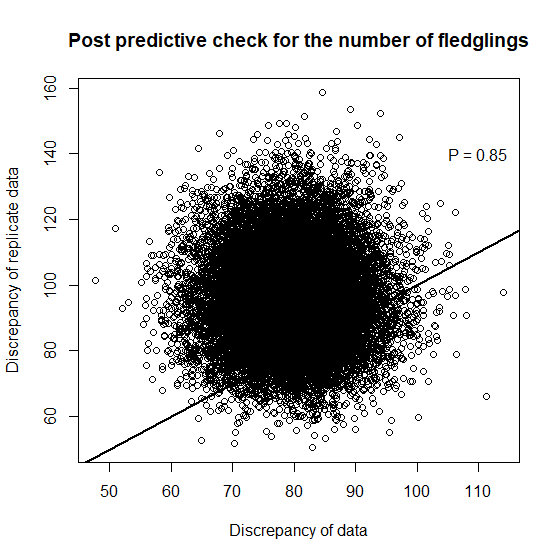


To access the goodness of fit of our **capture recapture models** we assessed the probabilities that the observed events (e.g. for adult breeders six possible events including uncertain state and not re-sighted) matched with events simulated from the categorical likelihoods used to model the observation process given the true states (Acker, 2017; Greenhill, Ward, & Sacks, 2011).

In addition, as the latent true states are partly informed by the data on the observed events (e.g. an observed event “failed breeder on short habitat” necessarily leads to the latent state “failed breeder on short habitat”), we also assessed the goodness of fit of the state processes by comparing the partly observed, partly estimated latent state of our model with a latent state 100% simulated from the categorical likelihoods used to model the state process given the model.

These probabilities were only calculated for the states and events following the first year of capture (as states and events at first sighting or before always match) and up to the last year of study for adult breeders.

The proportion of matching events were 0.991 (95% CI 0.986-0.995) for first year recruits and 0.980 (0.976-0.983) for adult breeders and the proportion of matching states were 0.849 (0.835-0.863) for recruits and 0.901 (0.896-0.906) for adult breeders.

Finally,we performed goodness of fit tests of our CMR model using the package R2Ucare (Gimenez et al., 2018) that suggested no lack of fit.

Jags code to assess goodness-of-fit is included in the script in Appendix 4. We used the same number of chains, burn in, thinning and samples as for the results reported in our study.


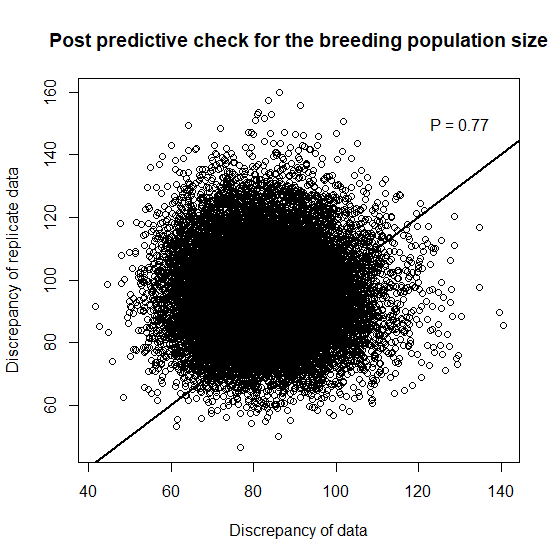


**Table A1**

Estimated arithmetic means and coefficient of temporal variance (for parameters with temporal variation) of demographic parameters for females with associated 95% credibility intervals. **n** refers to the proportion of occupied territory sites that are Short/Tall, **b** to breeding success, **f** to number of fledglings at successful sites, **φ** to apparent survival probabilities, **Ω** to the rate of apparent immigrant breeders relative to the total population, **ψ** to transition probabilities, **p** to re-sighting probability and **c** to state certainties. Age-specific parameters presented here were estimated without the state space model while the proportion of sites occupied and immigration rates were estimated from the integrated population model that did not consider age (as it would have resulted in a too small sample size for age specific female count data).

| **Parameter** | **Mean (95% BCI)** | **CV (95% BCI)** | **Parameter** | **Mean (95% BCI)** |
| --- | --- | --- | --- | --- |
| **n_,Short_** | 0.62 (0.60-0.64) | 0.15 (0.12-0.18) | **ψ*_fledg_* _Short_** | 0.42 (0.34, 0.51) |
| **n_,Tall_** | 0.38 (0.36-0.40) | 0.24 (0.19-0.29) | **ψ*_fledg_* _Tall_** | 0.62 (0.48, 0.75) |
| **b_Y,Short_** | 0.76 (0.72, 0.80) | 0.08 (0.01, 0.16) | **ψ*_success_* _Y_ _Short_** | 0.24 (0.15, 0.35) |
| **b_O,Short_** | 0.83 (0.81, 0.85) | 0.08 (0.05, 0.12) | **ψ*_success_* _O_ _Short_** | 0.19 (0.13, 0.26) |
| **b_Y,Tall_** | 0.64 (0.58, 0.69) | 0.07 (0.00, 0.19) | **ψ*_success_* _Y_ _Tall_** | 0.48 (0.31, 0.64) |
| **b_O,Tall_** | 0.70 (0.66, 0.74) | 0.16 (0.09, 0.22) | **ψ*_success_* _O_ _Tall_** | 0.49 (0.35, 0.64) |
| **f_Y,Short_** | 2.56 (2.42, 2.70) | 0.04 (0.00, 0.10) | **ψ*_fail_* _Y_ _Short_** | 0.01 (0.00, 0.10) |
| **f_O,Short_** | 2.82 (2.72, 2.92) | 0.02 (0.00, 0.06) | **ψ*_fail_* _O_ _Short_** | 0.17 (0.02, 0.41) |
| **f_Y,Tall_** | 2.28 (2.11, 2.46) | 0.08 (0.01, 0.17) | **ψ*_fail_* _Y_ _Tall_** | 0.76 (0.43, 0.97) |
| **f_O,Tall_** | 2.55 (2.40, 2.70) | 0.04 (0.00, 0.11) | **ψ*_fail_* _O_ _Tall_** | 0.55 (0.16, 0.91) |
| **φ*_fledg_* _Short_** | 0.09 (0.07, 0.10) | 0.45 (0.22, 0.64) | **p** | 0.89 (0.83, 0.93) |
| **φ*_fledg_* _Tall_** | 0.06 (0.05, 0.08) | 0.19 (0.01, 0.55) | **c** | 0.89 (0.86, 0.92) |
| **φ*_success_* _Y,Short_** | 0.48 (0.40, 0.56) | 0.08 (0.00, 0.24) | **c*_f_*** | 0.98 (0.95, 0.99) |
| **φ*_success_* _O,Short_** | 0.49 (0.44, 0.54) | 0.08 (0.00, 0.21) |  |  |
| **φ*_success_* _Y,Tall_** | 0.45 (0.34, 0.56) | 0.22 (0.02, 0.53) |  |  |
| **φ*_success_* _O,Tall_** | 0.45 (0.34, 0.56) | 0.15 (0.01, 0.42) |  |  |
| **φ*_fail_* _Y,Short_** | 0.31 (0.17, 0.48) | 0.40 (0.02, 0.96) |  |  |
| **φ*_fail_* _O,Short_** | 0.20 (0.11, 0.30) | 0.31 (0.01, 0.85) |  |  |
| **φ*_fail_* _Y,Tall_** | 0.27 (0.15, 0.42) | 0.54 (0.02, 1.15) |  |  |
| **φ*_fail_* _O,Tall_** | 0.17 (0.07, 0.30) | 0.77 (0.03, 1.77) |  |  |
| **Ω _Y, Short_** | 0.27 (0.23, 0.32) | 0.70 (0.51, 0.94) |  |  |
| **Ω _O, Tall_** | 0.20 (0.16, 0.23) | 0.64 (0.48, 0.84) |  |  |

**Figure A1**

Transient LTRE contributions represent the part of variation in growth rate explained by the variation of each parameter (mean and 95% credible intervals for the female-based model) **(a)**. Light green (white background) for Short sites and dark green (grey background) for Tall sites. For each parameter and habitat, values on the left are for Young breeders and on the right for Old breeders. Contributions can be summed to obtain, for example, the overall contribution of Short habitats (light green), Tall habitats (dark green) and apparent immigration (grey)

**
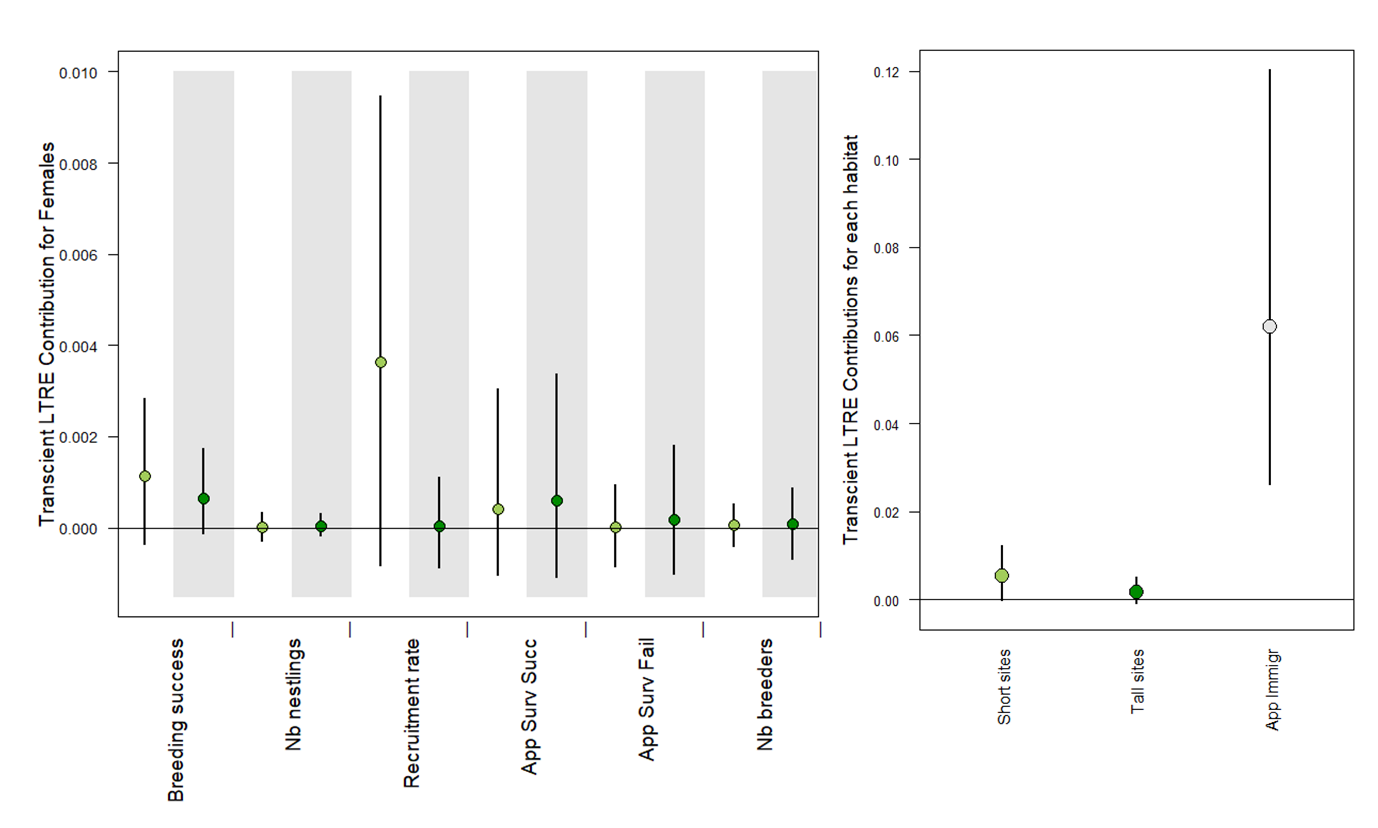
**

**Figure A2**

Average annual contributions of each habitat- and age-specific parameter to the change in population growth rate.


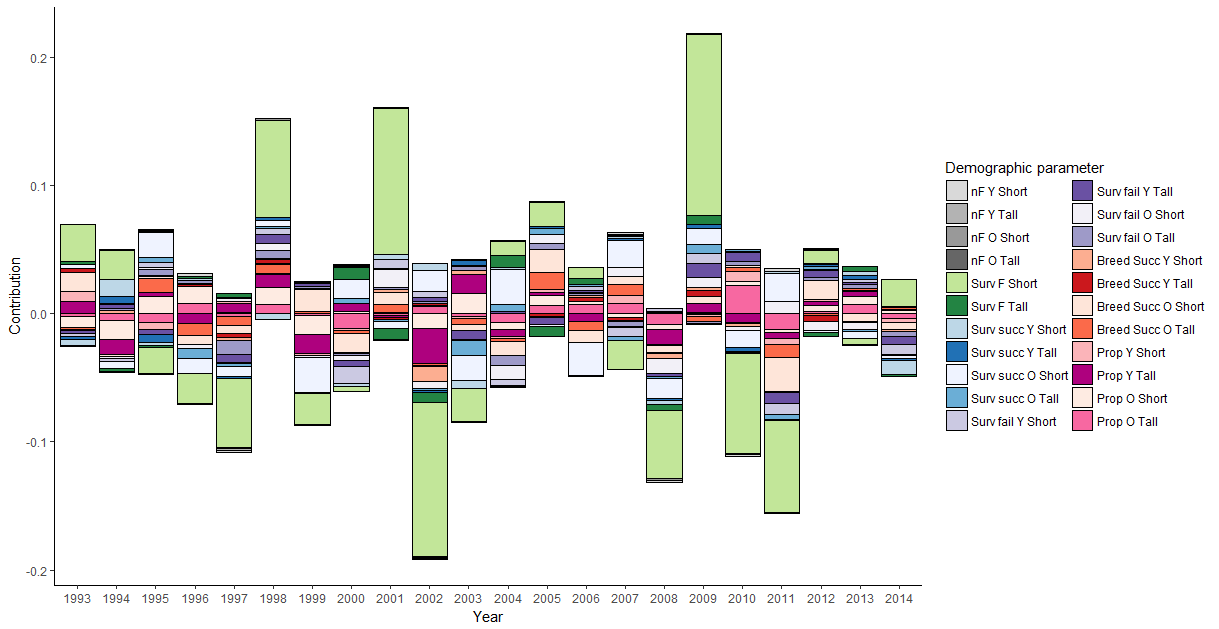


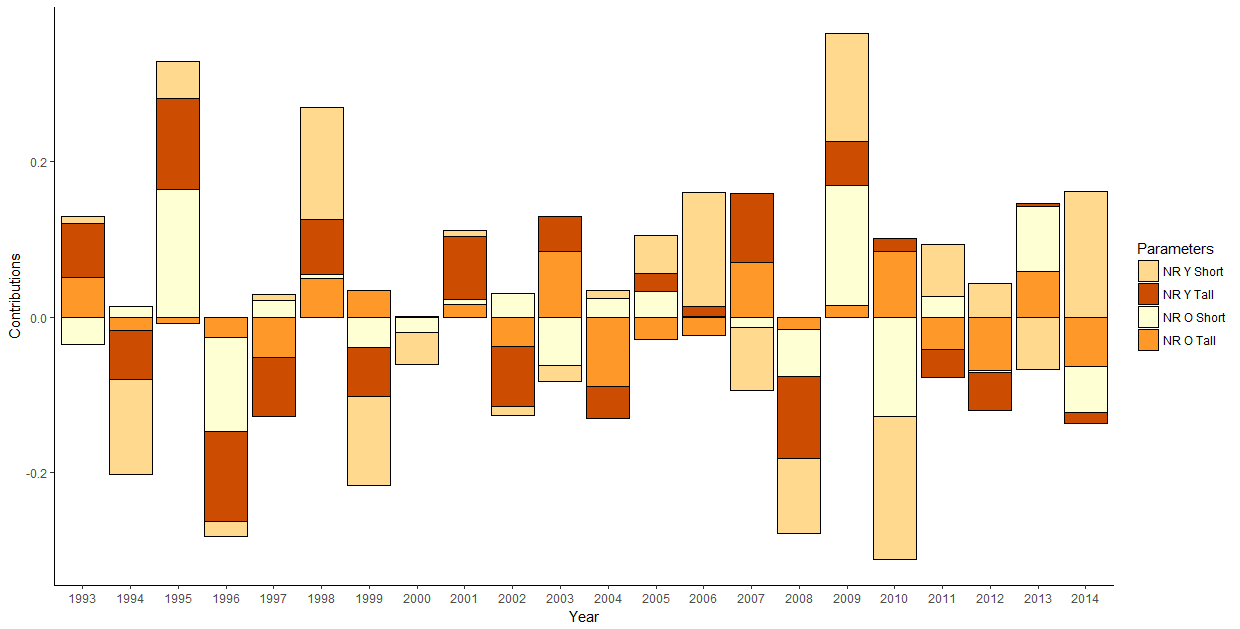


**Appendix A4: Jags code to fit the integrated population model (including code for post predictive checking)**

model{

# Per capita rate of increase per annum in population size

# Observation process

for(a in 1:2){

for(u in 1:2){

for (t in 1:nyears){

N.TOT[a,u,t]~dpois(N[a,u,t])

} #t

}#u

}#a

# Likelihood for juvenile and adult count data (state-space model)

# System process

for (t in 2:nyears){

Ntot[t]<-N[1,1,t]+N[1,2,t]+N[2,1,t]+N[2,2,t]

#one year old breeders in short habitats

N[1,1,t]<-Npsi.rec[1,2,t-1]+Npsi.rec[2,2,t-1]+(Nphi.rec[1,1,t-1]-Npsi.rec[1,1,t-1])+(Nphi.rec[2,1,t-1]-Npsi.rec[2,1,t-1])+Nimm[1,1,t]

#one year old breeders in tall habitats

N[1,2,t]<-Npsi.rec[1,1,t-1]+Npsi.rec[2,1,t-1]+(Nphi.rec[1,2,t-1]-Npsi.rec[1,2,t-1])+(Nphi.rec[2,2,t-1]-Npsi.rec[2,2,t-1])+Nimm[1,2,t]

#2+ years old breeders in short habitats

N[2,1,t]<-Npsipop[1,2,t-1]+Npsipop[2,2,t-1]+Npsipop[1,4,t-1]+Npsipop[2,4,t-1]+(Nphipop[1,1,t-1]-Npsipop[1,1,t-1])+(Nphipop[2,1,t-1]-Npsipop[2,1,t-1])+(Nphipop[1,3,t-1]-Npsipop[1,3,t-1])+(Nphipop[2,3,t-1]-Npsipop[2,3,t-1])+Nimm[2,1,t]

#2+ years old breeders in tall habitats

N[2,2,t]<-Npsipop[1,1,t-1]+Npsipop[2,1,t-1]+Npsipop[1,3,t-1]+Npsipop[2,3,t-1]+(Nphipop[1,2,t-1]-Npsipop[1,2,t-1])+(Nphipop[2,2,t-1]-Npsipop[2,2,t-1])+(Nphipop[1,4,t-1]-Npsipop[1,4,t-1])+(Nphipop[2,4,t-1]-Npsipop[2,4,t-1])+Nimm[2,2,t]

#Number of immigrants

Nimm[1,1,t]~dpois(mean.Nimm[1,1,t])

Nimm[1,2,t]~dpois(mean.Nimm[1,2,t])

Nimm[2,1,t]~dpois(mean.Nimm[2,1,t])

Nimm[2,2,t]~dpois(mean.Nimm[2,2,t])

}#t

###demographic stochasticity for the population level parameters

for (t in 2:nyears){

for(a in 1:2){

#number of survivors changing habitats (1 and 2 are successful breeders in short and tall habitats and 3 and 4 are failed breeders in short and tall habitats)

Npsipop[a,1,t-1]~ dbin(psipop[a,1,t-1],Nphipop[a,1,t-1])

Npsipop[a,2,t-1]~ dbin(psipop[a,2,t-1],Nphipop[a,2,t-1])

Npsipop[a,3,t-1]~ dbin(psipop[a,3,t-1],Nphipop[a,3,t-1])

Npsipop[a,4,t-1]~ dbin(psipop[a,4,t-1],Nphipop[a,4,t-1])

#number of survivors (1 and 2 are successful breeders in short and tall habitats and 3 and 4 are failed breeders in short and tall habitats)

Nphipop[a,1,t-1]~ dbin(phipop[a,1,t-1],NBS[a,1,t-1])

Nphipop[a,2,t-1]~ dbin(phipop[a,2,t-1],NBS[a,2,t-1])

Nphipop[a,3,t-1]~ dbin(phipop[a,3,t-1],(N[a,1,t-1]-NBS[a,1,t-1]))

Nphipop[a,4,t-1]~ dbin(phipop[a,4,t-1],(N[a,2,t-1]-NBS[a,2,t-1]))

}#a

}#t

for (t in 2:nyears){

for(u in 1:2){

for(a in 1:2){

#number of surviving fledgling changing habitats

Npsi.rec[a,u,t-1]~ dbin(psi.rec[u,t-1],Nphi.rec[a,u,t-1])

#number of surviving fledglings

Nphi.rec[a,u,t-1]~ dbin(phi.rec[u,t-1],Nnfledg[a,u,t-1])

#number of fledglings

Nnfledg[a,u,t-1] ~ dbinom(0.5,NnfledgMF[a,u,t-1])

NnfledgMF[a,u,t-1]<-nfl[a,u,t-1]+NBS[a,u,t-1]

nfl[a,u,t-1]~dpois(NBS[a,u,t-1]*(2*nfledg[a,u,t-1]-1))

#number of succesful sites

NBS[a,u,t-1]~dbin(BS[a,u,t-1],N[a,u,t-1])

}#a

}#u

}#t

####CMR ADULTS

# Likelihood of capture-recapture data

for (i in 1:nind){

latent.state[i,firstcap[i]]<-CMR[i,firstcap[i]]

# Observation process

for (t in (firstcap[i]+1):nyears){

CMR[i,t] ~ dcat(po[latent.state[i,t],i,t-1,]+0.000001)

# State process

latent.state[i,t] ~ dcat(ps[latent.state[i,t-1],i,t-1,]+0.000001)

}#t

}#i

####### State-transition and observation matrices for the multistate model

for (i in 1:nind){

for (t in firstcap[i]:(nyears-1)){

#### Define probabilities of state S(t+1) given State(t)

#States: 1: successful in short habitats

# 2:successful in tall habitats

# 3: failed in short

# 4: failed in tall

# 5:dead

#[initial state, indiv,t, new state]

ps[1,i,t,1]<-phi[i,1,t]*(1-psi[i,1,t])*BS[2,1,t+1]

ps[1,i,t,2]<-phi[i,1,t]*psi[i,1,t]*BS[2,2,t+1]

ps[1,i,t,3]<-phi[i,1,t]*(1-psi[i,1,t])*(1-BS[2,1,t+1])

ps[1,i,t,4]<-phi[i,1,t]*psi[i,1,t]*(1-BS[2,2,t+1])

ps[1,i,t,5]<-1-phi[i,1,t]

ps[2,i,t,1]<-phi[i,2,t]*psi[i,2,t]*BS[2,1,t+1]

ps[2,i,t,2]<-phi[i,2,t]*(1-psi[i,2,t])*BS[2,2,t+1]

ps[2,i,t,3]<-phi[i,2,t]*psi[i,2,t]*(1-BS[2,1,t+1])

ps[2,i,t,4]<-phi[i,2,t]*(1-psi[i,2,t])*(1-BS[2,2,t+1])

ps[2,i,t,5]<-1-phi[i,2,t]

ps[3,i,t,1]<-phi[i,3,t]*(1-psi[i,3,t])*BS[2,1,t+1]

ps[3,i,t,2]<-phi[i,3,t]*psi[i,3,t]*BS[2,2,t+1]

ps[3,i,t,3]<-phi[i,3,t]*(1-psi[i,3,t])*(1-BS[2,1,t+1])

ps[3,i,t,4]<-phi[i,3,t]*psi[i,3,t]*(1-BS[2,2,t+1])

ps[3,i,t,5]<-1-phi[i,3,t]

ps[4,i,t,1]<-phi[i,4,t]*psi[i,4,t]*BS[2,1,t+1]

ps[4,i,t,2]<-phi[i,4,t]*(1-psi[i,4,t])*BS[2,2,t+1]

ps[4,i,t,3]<-phi[i,4,t]*psi[i,4,t]*(1-BS[2,1,t+1])

ps[4,i,t,4]<-phi[i,4,t]*(1-psi[i,4,t])*(1-BS[2,2,t+1])

ps[4,i,t,5]<-1-phi[i,4,t]

ps[5,i,t,1]<-0

ps[5,i,t,2]<-0

ps[5,i,t,3]<-0

ps[5,i,t,4]<-0

ps[5,i,t,5]<-1

#######define probabilities of capture plus state attribution given state

#[new state,recapt, indiv,time, event (CMR)]

po[1,i,t,1]<-p[i,t]*pstu[i,t]

po[1,i,t,2]<-0

po[1,i,t,3]<-0

po[1,i,t,4]<-0

po[1,i,t,5]<-p[i,t]*(1-pstu[i,t])

po[1,i,t,6]<-1-p[i,t]

po[2,i,t,1]<-0

po[2,i,t,2]<-p[i,t]*pstu[i,t]

po[2,i,t,3]<-0

po[2,i,t,4]<-0

po[2,i,t,5]<-p[i,t]*(1-pstu[i,t])

po[2,i,t,6]<-1-p[i,t]

po[3,i,t,1]<-0

po[3,i,t,2]<-0

po[3,i,t,3]<-p[i,t]*pstu[i,t]

po[3,i,t,4]<-0

po[3,i,t,5]<-p[i,t]*(1-pstu[i,t])

po[3,i,t,6]<-1-p[i,t]

po[4,i,t,1]<-0

po[4,i,t,2]<-0

po[4,i,t,3]<-0

po[4,i,t,4]<-p[i,t]*pstu[i,t]

po[4,i,t,5]<-p[i,t]*(1-pstu[i,t])

po[4,i,t,6]<-1-p[i,t]

po[5,i,t,1]<-0

po[5,i,t,2]<-0

po[5,i,t,3]<-0

po[5,i,t,4]<-0

po[5,i,t,5]<-0

po[5,i,t,6]<-1

####### resighting probability and state certainty probability

p[i,t]<-mean.p

pstu[i,t]<-mean.pstu

}#t

}#i

#relationships for vital rate parameters

for(i in 1:nind){

for(u in 1:4){

for(t in firstcap[i]:(nyears-1)){

phi[i,u,t]<-phipop[age[i,t],u,t]

psi[i,u,t]<-psipop[age[i,t],u,t]

}#t

}#u

}#i

for(a in 1:2){

for(u in 1:4){

for(t in 1:(nyears-1)){

logit(phipop[a,u,t])<-eta.phi[a,u]+epsilon.phi[a,u,t]

logit(psipop[a,u,t])<-eta.psi[a,u]

epsilon.phi[a,u,t] ~ dnorm(0,tau.phi[a,u])

}#t

eta.phi[a,u] ~ dnorm(0,0.001) I(-15,15)

eta.psi[a,u] ~ dnorm(0,0.001) I(-15,15)

tau.phi[a,u]<-pow(sigma.phi[a,u],-2)

sigma.phi[a,u] ~ dunif(0,10)

}#u

}#a

#resighting probability

mean.p ~ dunif(0,1)

#prob of state certainty

mean.pstu ~ dunif(0,1)

########

###### CMR recruits

for (i in 1:nind.rec){

# State process

latent.state.rec[i,firstcap.rec[i]+1] ~ dcat(ps.rec[inivector.rec[i],i,])

# Observation process

juv.CMR[i,firstcap.rec[i]+1] ~ dcat(po.rec[latent.state.rec[i,firstcap.rec[i]+1],i,])

} #i

####### State-transition and observation matrices for the multistate model of recruits

for (i in 1:nind.rec){

#### Define probabilities of state S(t+1) given State(t)

#[initial state, indiv, new state]

ps.rec[1,i,1]<-(1-psi.rec[1,firstcap.rec[i]])*phi.rec[1,firstcap.rec[i]]

ps.rec[1,i,2]<-psi.rec[1,firstcap.rec[i]]*phi.rec[1,firstcap.rec[i]]

ps.rec[2,i,2]<-(1-psi.rec[2,firstcap.rec[i]])*phi.rec[2,firstcap.rec[i]]

ps.rec[2,i,1]<-psi.rec[2,firstcap.rec[i]]*phi.rec[2,firstcap.rec[i]]

ps.rec[1,i,3]<-(1-phi.rec[1,firstcap.rec[i]])

ps.rec[2,i,3]<-(1-phi.rec[2,firstcap.rec[i]])

##define probabilities of recapture and state attribution (t) given State(t)

#[new state, indiv, event (juv.CMR)]

po.rec[1,i,1]<-mean.p*pstu.rec[firstcap.rec[i]]

po.rec[1,i,2]<-0

po.rec[1,i,3]<-mean.p*(1-pstu.rec[firstcap.rec[i]])

po.rec[1,i,4]<-1-mean.p

po.rec[2,i,1]<-0

po.rec[2,i,2]<-mean.p*pstu.rec[firstcap.rec[i]]

po.rec[2,i,3]<-mean.p*(1-pstu.rec[firstcap.rec[i]])

po.rec[2,i,4]<-1-mean.p

po.rec[3,i,1]<-0

po.rec[3,i,2]<-0

po.rec[3,i,3]<-0

po.rec[3,i,4]<-1

}#i

#relationships for vital rate parameters of recruits

for(u in 1:2){

for(t in 1:(nyears.rec-1)){

logit(phi.rec[u,t])<-mu.rec.phi[u]+epsilon.rec.phi[u,t]

logit(psi.rec[u,t])<-mu.rec.psi[u]

epsilon.rec.phi[u,t] ~ dnorm(0,tau.rec.phi[u])

} # time

mu.rec.phi[u] ~ dnorm(0,0.001) I(-10,10)

mu.rec.psi[u] ~ dnorm(0,0.001) I(-10,10)

tau.rec.phi[u]<-pow(sigma.rec.phi[u],-2)

sigma.rec.phi[u] ~ dunif(0,10)

} # site

#prob of state certainty for 1 year old

for(t in 1:(nyears.rec-1)){

pstu.rec[t]<-mean.pstu.rec

}

mean.pstu.rec ~ dunif(0,1)

### Initial population sizes and priors

Ntot[1]<-N[2,2,1]+N[2,1,1]+N[1,2,1]+N[1,1,1]

nyflh1 ~ dnorm(5, 0.01) I(1,200)

N[1,1,1] <- round(nyflh1)

nyflh2 ~ dnorm(11, 0.01) I(1,200)

N[1,2,1] <- round(nyflh2)

noflh1 ~ dnorm(24, 0.01) I(1,200)

N[2,1,1] <- round(noflh1)

noflh2 ~ dnorm(9, 0.01) I(1,200)

N[2,2,1] <- round(noflh2)

#these values are arbitrary and never used

Nimm[1,1,1]<-1

Nimm[1,2,1]<-1

Nimm[2,1,1]<-1

Nimm[2,2,1]<-1

for(t in 2:nyears){

mean.Nimm[1,1,t]~dunif(-5,20)

mean.Nimm[1,2,t]~dunif(-5,20)

mean.Nimm[2,2,t]~dunif(-5,20)

mean.Nimm[2,1,t]~dunif(-5,20)

} # time

#Likelihood for the number of fledglings from successful sites

#note that rho.fledg corresponds to the total number of fledglings from successful sites minus the total number of successful sites (in order to use a Poisson distribution)

for(a in 1:2){

for(u in 1:2){

for(t in 1:nyears){

rho.fledg[a,u,t]~dpois(fledg.sample.TOT[a,u,t]*(2*nfledg[a,u,t]-1))

#likelihood for Breeding Success

BS.success.TOT[a,u,t] ~ dbin(BS[a,u,t],BS.sample.TOT[a,u,t])

BS[a,u,t]<-exp(logit.BS[a,u,t])/(1+exp(logit.BS[a,u,t]))

nfledg[a,u,t]<-exp(log.nfledg[a,u,t])

logit.BS[a,u,t]<-mu.BS[a,u]+epsilon.BS[a,u,t]

log.nfledg[a,u,t]<-mu.nfledg[a,u]+epsilon.nfledg[a,u,t]

epsilon.BS[a,u,t] ~ dnorm(0,tau.BS[a,u])

epsilon.nfledg[a,u,t] ~ dnorm(0,tau.nfledg[a,u])

} # time

tau.BS[a,u]<-pow(sigma.BS[a,u],-2)

tau.nfledg[a,u]<-pow(sigma.nfledg[a,u],-2)

sigma2.BS[a,u]<-pow(sigma.BS[a,u],2)

sigma2.nfledg[a,u]<-pow(sigma.nfledg[a,u],2)

sigma.BS[a,u] ~ dunif(0,10)

sigma.nfledg[a,u] ~ dunif(0,10)

mu.BS[a,u] ~ dnorm(0,0.001)

mu.nfledg[a,u] ~ dnorm(0,0.001)

} # site

} # age

############################

#POST PREDICTIVE CHECKS#####

############################

####evaluate fit for BS

for(a in 1:2){

for(u in 1:2){

for(t in 1:nyears){

eval.BS[a,u,t]<-BS[a,u,t]*BS.sample.TOT[a,u,t]

E.BS[a,u,t]<-pow((BS.success.TOT[a,u,t]-eval.BS[a,u,t]),2)/(eval.BS[a,u,t]*(1-BS[a,u,t])+0.01)

####

BS.success.new[a,u,t]~dbin(BS[a,u,t],BS.sample.TOT[a,u,t])

E.BS.new[a,u,t]<-pow((BS.success.new[a,u,t]-eval.BS[a,u,t]),2)/(eval.BS[a,u,t]*(1-BS[a,u,t])+0.01)

}#t

}#u

}#a

fit.BS<-sum(E.BS[,,])

fit.BS.new<-sum(E.BS.new[,,])

####evaluate fit for fledglings

for(a in 1:2){

for(u in 1:2){

for(t in 1:nyears){

eval.rho.fledg[a,u,t]<-fledg.sample.TOT[a,u,t]*(2*nfledg[a,u,t]-1)

E.rho.fledg[a,u,t]<-pow((rho.fledg[a,u,t]-eval.rho.fledg[a,u,t]),2)/(eval.rho.fledg[a,u,t]+0.01)

rho.fledg.new[a,u,t]~dpois(fledg.sample.TOT[a,u,t]*(2*nfledg[a,u,t]-1))

E.rho.fledg.new[a,u,t]<-pow((rho.fledg.new[a,u,t]-eval.rho.fledg[a,u,t]),2)/(eval.rho.fledg[a,u,t]+0.01)

}#t

}#u

}#a

fit.fledg<-sum(E.rho.fledg[,,])

fit.fledg.new<-sum(E.rho.fledg.new[,,])

####

####evaluate fit for Ncount

for(a in 1:2){

for(u in 1:2){

for(t in 1:nyears){

eval.N[a,u,t]<-N[a,u,t]

E.N[a,u,t]<-pow((N.TOT[a,u,t]-eval.N[a,u,t]),2)/(eval.N[a,u,t]+0.01)

N.TOT.new[a,u,t]~dpois(N[a,u,t])

E.N.new[a,u,t]<-pow((N.TOT.new[a,u,t]-eval.N[a,u,t]),2)/(eval.N[a,u,t]+0.01)

}#t

}#u

}#a

fit.N<-sum(E.N[,,])

fit.N.new<-sum(E.N.new[,,])

####evaluate fit CMR fledglings

CP.mean.rec<-sum(CP.rec)/sum(CP.tot.rec)

CP.state.mean.rec<-sum(CP.state.rec)/sum(CP.state.tot.rec)

for (i in 1:nind.rec){

CP.state.tot.rec[i]<-1

CP.tot.rec[i]<-1

}

for (i in 1:nind.rec){

juv.CMR.new[i] ~ dcat(po.rec[latent.state.rec[i,firstcap.rec[i]+1],i,])

CP.rec[i] <- ifelse(juv.CMR.new[i]==juv.CMR[i,firstcap.rec[i]+1],1,0)

CP.state.rec[i] <- ifelse(latent.state.rec.new[i]==latent.state.rec[i,firstcap.rec[i]+1],1,0)

latent.state.rec.new[i] ~ dcat(ps.rec[inivector.rec[i],i,])

} #i

####

####evaluate fit CMR adults

CP.mean<-sum(CP.sum.ind)/sum(CP.sum.tot.ind)

CP.state.mean<-sum(CP.state.sum.ind)/sum(CP.sum.tot.ind)

for(i in 1:nind){

CP.sum.ind[i]<-sum(CP[i,(firstcap[i]+1):nyears])

CP.state.sum.ind[i]<-sum(CP.state[i,(firstcap[i]+1):nyears])

CP.sum.tot.ind[i]<-sum(CP.state.tot[i,(firstcap[i]+1):nyears])

}

for (i in 1:nind){

for(t in 1:nyears){

CP.state.tot[i,t]<-1

CP.tot[i,t]<-1

}

}

for (i in 1:nind){

for (t in (firstcap[i]+1):nyears){

# Observation process: draw O(t) given S(t)

CMR.new[i,t] ~ dcat(po[latent.state[i,t],i,t-1,]+0.000001)

CP[i,t] <- ifelse(CMR.new[i,t]==CMR[i,t],1,0)

latent.state.new[i,t] ~ dcat(ps[latent.state[i,t-1],i,t-1,]+0.000001)

CP.state[i,t] <- ifelse(latent.state.new[i,t]==latent.state[i,t],1,0)

}#t

}#i

############################ end of model fitting section

############################

}#model

# Bundle data

jags.data <- list( inivector.rec=inivector.rec,rho.fledg=rho.fledg,nyears.rec = dim(juv.CMR)[2], nind.rec = dim(juv.CMR)[1],firstcap.rec=firstcap.rec,latent.state.rec=latent.state.rec,juv.CMR=juv.CMR,nind = dim(CMR)[1], firstcap=firstcap,latent.state=latent.state,CMR=CMR,age=age,N.TOT=N.TOT,BS.success.TOT=BS.success.TOT,BS.sample.TOT=BS.sample.TOT,fledg.sample.TOT=fledg.sample.TOT,nyears=dim(CMR)[2])

###initial values

inits<-function(){list(mu.BS=array(runif(4, 1/(1+exp(-0)), 1/(1+exp(-1))),dim=c(2,2)),

epsilon.BS=array(runif(4*(dim(BS.success.TOT)[3]), 1/(1+exp(-0)), 1/(1+exp(-1))),dim=dim(BS.success.TOT)),

sigma.BS=array(runif(4, 0.1, 10),dim=c(2,2)),

eta.phi=array(c(1/(1+exp(-0.52)), 1/(1+exp(-0.52)),1/(1+exp(-0.46)),1/(1+exp(-0.46)),1/(1+exp(-0.45)),1/(1+exp(-0.45)),1/(1+exp(-0.45)),1/(1+exp(-0.45))),dim=c(2,4)),

eta.psi=array(c(1/(1+exp(-0.15)), 1/(1+exp(-0.15)),1/(1+exp(-0.30)),1/(1+exp(-0.30)),1/(1+exp(-0.20)),1/(1+exp(-0.20)),1/(1+exp(-0.35)),1/(1+exp(-0.35))),dim=c(2,4)),

mean.p=0.98,

mean.pstu=runif(1,0.6,0.99),

mu.rec.phi=runif(2, 1/(1+exp(-0.1)), 1/(1+exp(-0.3))),

mu.rec.psi=runif(2, 1/(1+exp(-0.2)), 1/(1+exp(-0.7))),

mean.pstu.rec=runif(1,0.6,0.99)

)

}

ni <- 30000

nt <- 3

nb <- 10000

nc <- 3

IPMwheatears<-jags(jags.data,inits=inits,parameters,model.file="IPMwheatears.jags",n.chains=nc,n.thin=nt,n.iter=ni,n.burnin=nb,working.directory=getwd())

**Appendix references**

Acker, P. (2017). *Life-history decisions of larids in spatio-temporally varying habitats: where and when to breed* (PhD Thesis). Université de Toulouse, Université Toulouse III-Paul Sabatier.

Gelman, A., Meng, X.-L., & Stern, H. (1996). Posterior predictive assessment of model fitness via realized discrepancies. *Statistica Sinica*, 733–760.

Gimenez, O., Lebreton, J.-D., Choquet, R., & Pradel, R. (2018). R2ucare: An r package to perform goodness-of-fit tests for capture–recapture models. *Methods in Ecology and Evolution*.

Greenhill, B., Ward, M. D., & Sacks, A. (2011). The separation plot: A new visual method for evaluating the fit of binary models. *American Journal of Political Science*, *55*(4), 991–1002.

Koons, D. N., Arnold, T. W., & Schaub, M. (2017). Understanding the demographic drivers of realized population growth rates. *Ecological Applications*.

Koons, D. N., Iles, D. T., Schaub, M., & Caswell, H. (2016). A life‐history perspective on the demographic drivers of structured population dynamics in changing environments. *Ecology Letters*, *19*(9), 1023–1031.
